# Transcriptome signatures for the identification of bevacizumab responders in ovarian cancer

**DOI:** 10.1186/s13073-026-01727-6

**Published:** 2026-08-05

**Authors:** Olga Zolotareva, Karen Legler, Olga Tsoy, Anna Esteve, Alexey Sergushichev, Vladimir Sukhov, Jan Baumbach, Kathrin Eylmann, Minyue Qi, Malik Alawi, Stefan Kommoss, Barbara Schmalfeldt, Leticia Oliveira-Ferrer

**Affiliations:** 1 Institute for Computational Systems Biomedicine, University of Hamburg, Hamburg, Germany; 2 Institute of Clinical Molecular Biology (IKMB), Kiel University and University Medical Center Schleswig-Holstein, Kiel, Germany; 3 Data Science in Systems Biology, TUM School of Life Sciences, Technical University of Munich, Freising, Germany; 4 Department of Gynecology, University Medical Center Hamburg-Eppendorf, Hamburg, Germany; 5 Computer Science Department, Vrije Universiteit Amsterdam, Amsterdam, the Netherlands; 6 Medical Oncology Service, Catalan Institute of Oncology (ICO), B-ARGO/CARE Program, Germans Trias i Pujol Research Institute (IGTP), Barcelona, Spain; 7 Department of Pathology and Immunology, Washington University in St. Louis School of Medicine, St. Louis, MO USA; 8 Computational BioMedicine Lab, University of Southern Denmark, Odense, Denmark; 9 Bioinformatics Core, University Medical Center Hamburg-Eppendorf, Hamburg, Germany; 10 Department of Gynecology and Obstetrics, University Hospital Tuebingen, Tuebingen, Germany; 11 Department of Gynecology and Obstetrics, Diak Klinikum Schwaebisch Hall, Diakoniestrasse 10, Schwaebisch Hall, 74523 Germany

**Keywords:** Ovarian cancer, Transcriptome, Differential expression, Expression signature, Bevacizumab response, Biclustering

## Abstract

**Background:**

Bevacizumab is widely used as an anti-angiogenic maintenance therapy in ovarian cancer; however, there are currently no validated clinical criteria to guide patient selection for its use.

**Methods:**

To satisfy the urgent need for bevacizumab response biomarkers, we created a novel RNA-seq dataset (*n* = 244) and applied unsupervised and supervised machine learning to identify expression signatures associated with benefit from adding bevacizumab to standard treatment and validated our findings using a previously published microarray dataset (*n* = 377). Additionally, we validated the existence of the discovered signatures using RNA-seq data from the TCGA-OV cohort (*n* = 426) and performed public expression data mining to provide a biological interpretation of the prioritized signature.

**Results:**

Among expression signatures reproducibly detected in independent datasets, one was prioritized as a potential predictive biomarker for bevacizumab benefit. Further stratified analysis revealed that over-expression of this signature was associated with improved overall survival in patients who received bevacizumab in addition to standard chemotherapy in both novel (HR = 0.41, 95% CI: (0.23–0.74), adj.*p*-value = 0.008) and previously published cohorts (HR = 0.51, 95% CI: (0.34–0.75), adj.*p*-value = 0.003), while no significant survival benefit from bevacizumab was observed in patients negative for this signature. We hypothesize that this signature may be associated with stemness-like features, possibly driven by *CTCFL*. In addition, we identified several other signatures reproducible in independent datasets and not related to known molecular subtypes of ovarian cancer, which may also represent biomarker candidates and require further validation in additional RNA-seq data.

**Conclusions:**

We identified a previously undescribed expression signature with potential predictive value for bevacizumab benefit, and revealed transcriptional heterogeneity of ovarian cancer that extends beyond current molecular classifications. Given the high heterogeneity of ovarian cancer and that the novel signature only partially explains variation in survival outcomes under bevacizumab treatment, larger RNA-seq datasets are required to further improve predictive models.

**Supplementary Information:**

The online version contains supplementary material available at 10.1186/s13073-026-01727-6.

## Background

Ovarian cancer is the second leading cause of death from gynecological cancer with an overall 5-year survival rate of less than 50%. This high mortality is mainly due to the lack of effective screening tools, leading to 75% of women being diagnosed with advanced stages and a high recurrence rate [1]. Standard of care consists of cytoreductive surgery followed by adjuvant platinum-based plus taxane therapy and maintenance therapy with the antiangiogenic compound bevacizumab and/or a PARP inhibitor [2].

Bevacizumab is an antibody against vascular endothelial growth factor (VEGF), a key factor in the regulation of angiogenesis. In cancer, VEGF inhibition slows or prevents the growth of blood vessels that supply the tumor with oxygen and nutrients, leading to tumor necrosis. Several studies have shown that the addition of bevacizumab during first-line therapy, followed by 10 months of monotherapy with bevacizumab, leads to a significant extension of progression-free survival (PFS) by 4 months [3–5]. It is important to point out that the use of bevacizumab in cancer treatment leads to potential side effects, such as hypertension and gastrointestinal bleeding/perforation, and the decision to include it in a treatment plan is usually based on an assessment of the potential benefits versus risks for the patient. In this context, the identification of patients who will benefit from antiangiogenic therapy is crucial, and many efforts have been made over the last decade to identify biomarkers of response to bevacizumab in ovarian cancer.

In the context of the ICON7 trial, several serum biomarkers have been tested: a discriminatory signature comprising mesothelin, *FLT4*, *AGP*, and *CA-125* has been described to potentially identify those patients with epithelial ovarian cancer (EOC) more likely to benefit from the antiangiogenic therapy [6], and also the combined values of serum Ang1 and Tie2 levels has been proposed as predictive biomarkers for improved PFS in bevacizumab-treated patients with ovarian cancer [7]. Additionally, serum *VEGF-A* or *VEGF-C* levels have been identified in other cohorts to be significantly associated with response to bevacizumab therapy [8, 9]. Also, YKL-40, a proangiogenic glycoprotein secreted by cancer and inflammatory cells as a consequence of VEGF inhibition, is associated with improved outcomes in patients with chemotherapy-refractory advanced ovarian cancer treated with bevacizumab [10].

In the context of the GOG-0218 clinical trial [11], five candidate tumor biomarkers (*VEGF-A, CD31*, neuropilin-1, *MET, VEGFR-2*) were assessed by immunohistochemistry in an extensive population (*n* = 980), but only higher microvessel density (measured by *CD31*) showed predictive value for PFS. Tumor *VEGF-A* was not predictive for PFS but showed potential predictive value for overall survival (OS) using a third-quartile cutoff for high *VEGF-A* expression [12]. In contrast, *VEGF-A165b*, a *VEGF-A* splice variant, was analyzed in a tissue microarray of the ICON7 trial, suggesting that bevacizumab may improve the prognosis of patients with advanced ovarian cancer with low expression of this *VEGFA* isoform [13]. In the same cohort, immunofluorescence staining for co-localised *c-MET* and *VEGFR-2* and genotyping of germline DNA from peripheral blood leukocytes for *VEGFA* and *VEGFR-2* SNPs were performed. Here, high *c-MET/VEGFR-2* co-localisation on tumor tissue and the *VEGFR-2* rs2305945 G/G variant, which may be biologically related, were associated with worse survival outcomes in bevacizumab-treated women diagnosed with EOC [14]. In addition, Volk et al. showed that patients with high *Ang-2* expression benefit significantly from therapy with bevacizumab [15].

At the gene expression level, data from the ICON7 and the MITO16A-ManGo-OV2 phase IV trial have been evaluated using DASL gene expression arrays and qRT-PCR, respectively, for the identification of signatures of bevacizumab response [16, 17]. Kommoss et al. found that molecular subtypes of ovarian carcinoma with the poorest survival (proliferative and mesenchymal) show a higher benefit from treatment including bevacizumab. Califano et al. found high *miR-484* expression to be associated with longer progression-free and overall survival. Interestingly, the combined expression of *miR-484* and its target *VEGFB* identified a subset of patients that might mostly benefit from bevacizumab treatment.

Since previously published biomarker candidates predictive of bevacizumab response have not yet been validated using independent datasets, their clinical relevance remains uncertain. To bridge this gap and identify reproducible biomarker candidates enabling personalization of bevacizumab therapy in EOC, in the present study, we generated a novel transcriptome dataset, the UKE cohort, comprising 244 ovarian tumor samples obtained from 212 patients treated at Universitätsklinikum Hamburg-Eppendorf. Complete clinical information was available for 181 patients, of whom 114 were treated only with platinum-based therapy and 67 additionally received bevacizumab as maintenance therapy. We utilized unsupervised and supervised machine learning to discover candidate expression signatures with potential predictive significance in bevacizumab-treated EOC. A previously published EOC cohort DASL [16] with similar patient characteristics and treatment settings was used as a validation dataset. Additionally, the existence of the candidate expression signatures discovered in the UKE and DASL data, and their relationships with known molecular subtypes of EOC, was confirmed using another publicly available RNA-seq-based dataset, TCGA-OV.

## Methods

### UKE and DASL cohorts

The UKE cohort was established at the Department of Gynecology, University Medical Center Hamburg Eppendorf (UKE), Hamburg, Germany. Cryopreserved tumor tissue samples (*n* = 244) obtained from 212 patients with peritoneal, fallopian tube and ovarian carcinoma treated at this center between 2009 and 2017 were available in the gynaecological biobank and were included in this cohort. All patients gave written informed consent for biomaterial to be collected and for access to their clinical records. The procedure was approved in advance by the Ethics Committee (Ethik-Kommission der Ärztekammer Hamburg, No. 190504). Patient data were followed from the date of initial diagnosis until 2022. Detailed information on the RNA isolation procedure and RNAseq analysis is provided below. The characteristics of the entire reduced UKE cohort used in the current study are shown in Table 1. The DASL cohort has been described previously [16]. Briefly, paraffin-embedded tissue was available from 423 patients with primary ovarian, fallopian tube or primary peritoneal cancer from the AGO-OVAR11 trial who were initially included in this cohort. For further analysis, 380 samples with both expression and clinical data available were included.

**Table 1:** The baseline characteristics of the UKE cohort

		**Bevacizumab (*n* = 67)**	**Standard (*n* = 114)**	**Total (*n* = 181)**
FIGO				
	I	0	2 (1.8%)	2 (1.1%)
	II	0	5 (4.4%)	5 (2.8%)
	III	54 (80.6%)	83 (72.8%)	137 (75.7%)
	IV	13 (19.4%)	24 (21.1%)	37 (20.4%)
Grading				
	low grade (G1/G2)	4 (6.0%)	19 (16.7%)	23 (12.7%)
	high grade	61 (91.0%)	95 (83.3%)	156 (86.2%)
	unknown	2 (3.0%)	0	2 (1.1%)
Stage				
	pT1-pT2	5 (7.5%)	6 (5.3%)	11 (6.1%)
	pT3a-pT3b	9 (13.4%)	7 (6.1%)	16 (8.8%)
	pT3c	51 (76.1%)	100 (87.7%)	151 (83.4%)
	unknown	2 (3.0%)	1 (0.9%)	3 (1.7%)
pN				
	N0	12 (17.9%)	24 (21.1%)	36 (19.9%)
	N1	43 (64.2%)	65 (57.0%)	108 (59.7%)
	Nx	12 (17.9%)	25 (21.9%)	37 (20.4%)
Histological Type				
	serous	62 (92.5%)	107 (93.9%)	169 (93.4%)
	other or unknown	5 (7.5%)	7 (6.1%)	12 (6.6%)
Surgery outcome				
	no macroscopically visible tumor	39 (58.2%)	73 (64.0%)	112 (61.9%)
	tumor ≤ 1 cm	14 (20.9%)	27 (23.7%)	41 (22.7%)
	tumor > 1 cm	14 (20.9%)	14 (12.3%)	28 (15.5%)
Age at diagnosis	years, mean (CI95%)	59.4 (56.4–62.4)	60.3 (58.3–62.2)	59.9 (58.3—61.6)
Median follow-up	months, median	41.0	41.5	41.0
Events at median follow-up	death	35 (52.2%)	85 (74.6%)	120 (66.3%)
	recurrence	55 (82.1%)	93 (81.6%)	148 (81.8%)

### RNA isolation from ovarian cancer tissue and RNAseq analysis

RNA isolation from 244 tumor tissue samples corresponding to 212 ovarian cancer patients was performed as previously described [18, 19]. Briefly, tissue samples obtained during cytoreductive surgery were immediately stored in liquid nitrogen as fresh frozen samples. Haematoxylin–eosin-stained cryo-sections were performed to evaluate the histological characteristics and tumor content of each sample. Subsequently, 20–30 cryosections (approximately 16 µm) were disintegrated using the Precellys homogeniser (WVR International GmbH, Darmstadt, Germany) and RNA was extracted using the RNAeasy Kit (Qiagen GmbH, Hilden, Germany), according to the manufacturer’s instructions. RNA quantity and integrity were assessed using a Bioanalyzer device (Agilent, Santa Clara, CA, USA). RNA sequencing was performed by BGI Genomics (Shenzhen, China) using the DNBseq™ Technology Platform. Sequence reads were processed with fastp (v0.23.2) to remove sequences originating from sequencing adapters and sequences of low quality (Phred quality score below 15) [20]. Reads were then aligned to the human reference assembly (GRCh38.106) using STAR (v2.7.10a) [21]. Gene-level raw read counts were normalized using the median ratio method [22] implemented in DESeq2 (v1.34.0) [23] R package and log2(x + 1) transformed. Genes with 15 or more normalized counts in less than 10 samples were considered weakly or infrequently expressed and were excluded from further analysis.

### Expression data

Transcriptome data and corresponding clinical and histopathological information from three independently created ovarian cancer patient cohorts were analyzed in this study. For two of them, data was publicly available: DASL (GSE140082) [16] and TCGA-OV [24].

Normalized and log-transformed gene-level expressions from the DASL cohort comprising 380 samples preprocessed as described in Kommoss et al. 2017 [16] were provided by Stefan Kommoss. Three samples were excluded from the analysis because information on tumor stage or operation result was missing.

RSEM-normalized and log2-transformed gene-level read counts and patient data including OS and PFS time and events, tumor stages for TCGA-OV cohort [24] were obtained from XENA [25] TCGA Pan-Cancer (PANCAN) data hub (https://xenabrowser.net/datapages/?cohort=TCGA%20Pan-Cancer%20(PANCAN)).

The UKE dataset comprises 244 tumor samples obtained from 212 individuals diagnosed with EOC. Gene-level read counts and sample metadata data are available in Additional file 1: Table S1 of this article. Normalized and log2(x + 1) transformed expression profiles of all 244 samples were used in biclustering analysis to facilitate the detection of infrequent sample subsets, but only 181 patients treated with standard chemotherapy with or without bevacizumab, for whom complete clinical information was available, were taken into account in survival analysis. Patients who received neoadjuvant or treatment different from standard chemotherapy or bevacizumab (*n* = 18), had recurrent EOC (*n* = 6), or for whom information on survival, therapy, age at diagnosis, FIGO and/or residual tumor after surgery was not available (*n* = 4) were excluded from survival analysis.

### Identification of known molecular subtypes

Molecular subtypes were determined using a random forest-based classifier implemented in *consensusOV* [26] R package v1.24.0. This consensus classifier trained on tumors from multiple independent datasets, concordantly subtyped by three previously published classifiers developed by Konecny et al. [27], Verhaak et al. [28], and Helland et al. [29], and demonstrated improved robustness compared to individual classifiers. Molecular subtypes were assigned using the *get.subtypes()* function applied to normalized, log2(x + 1)-transformed and standardized expression data with default parameters. Consensus molecular subtypes predicted for the DASL and TCGA-OV cohorts were similar to previously published classifications (Additional file 1: Table S2A-C). Additionally, to ensure that the predicted molecular subtypes consistently express biomarkers described in the literature, based on the lists used by Verhaak et al. [28] and Talhouk et al. [30] we compiled a list of 98 subtype-specific biomarkers (18 for proliferative, 27 for mesenchymal, 16 for differentiated, and 37 for immunoreactive; Additional file 1: Table S2D) and verified their expression in three cohorts UKE, DASL and TCGA-OV (Additional file 2: Fig. S1).

### Biclustering for unsupervised patient stratification

**Fig. 1:**
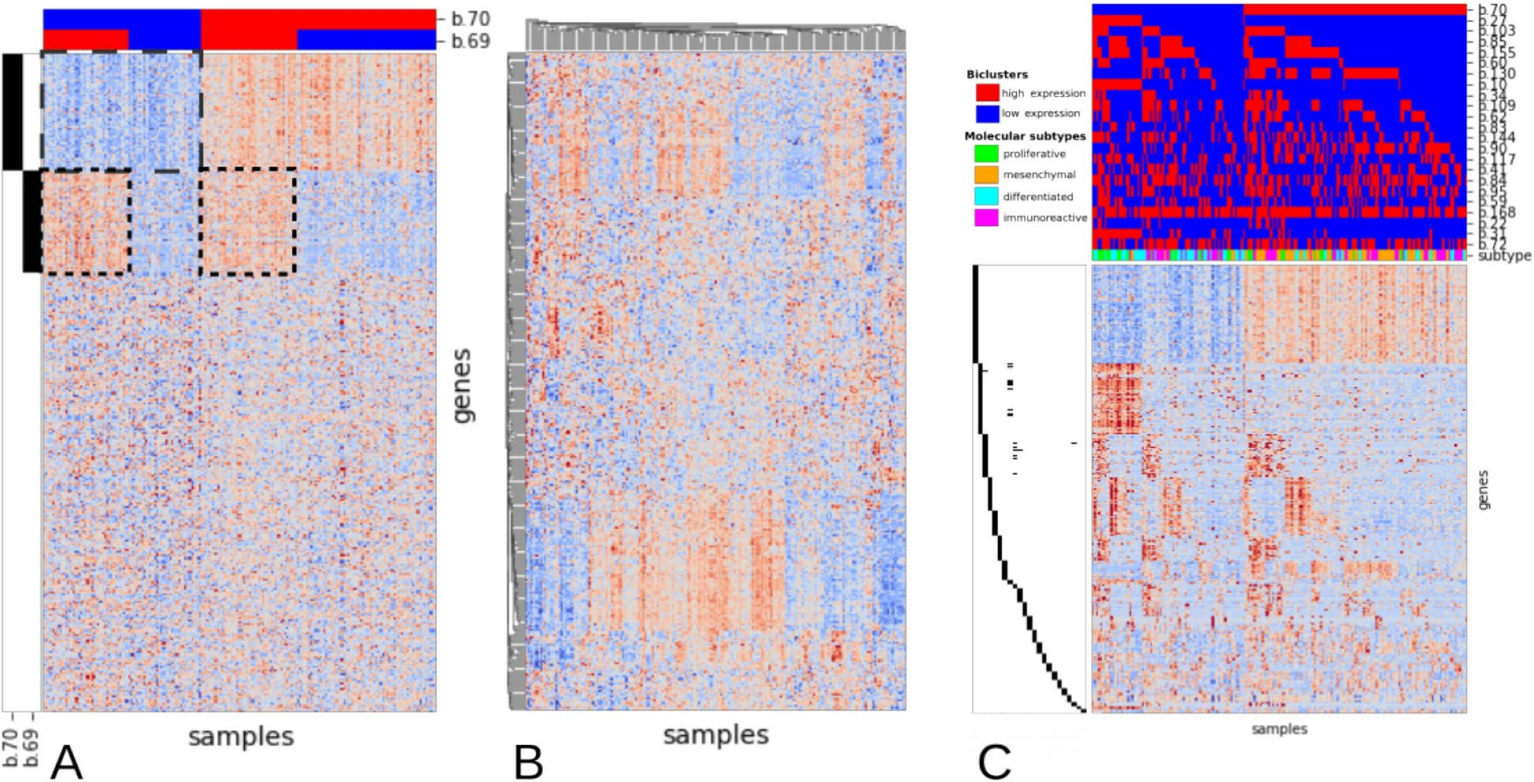
Biclustering and clustering analysis results on the example of the UKE dataset. For illustration purposes, 109 genes from two biclusters identified by UnPaSt and 218 randomly chosen genes are shown in panes **A** and **B**. **A** Two biclusters identified by UnPaSt are highlighted with dashed frames. Gene membership in biclusters is shown with black bars on the left margin of the heatmap. Colorbars on the top of the heatmap show whether the bicluster genes are up- or down-regulated in each sample. Although a bicluster always divides samples into two subpopulations, we refer to a smaller subpopulation as “bicluster” and to a larger one as “background”. If a bicluster divides all patients into two equal-sized subsets, the subset with higher expression is assigned to the “bicluster” group. Based on the direction of the expression pattern, up-regulated (e.g. bicluster b.69), down-regulated (e.g. bicluster b.70) biclusters can be distinguished. **B** The results of hierarchical clustering of the same samples and genes as in panel **A**, using average linkage and Euclidean distance metrics. **C** 23 biclusters identified in the UKE datasets which were replicated in DASL. Expression of 264 genes in 181 samples from the UKE dataset is shown, biclusters are ordered by the number of genes decreasing. The colorbar on the upper border of the heatmap shows molecular subtypes determined by *consensusOV* classifier (green—proliferative, orange—mesenchymal, cyan—differentiated, magenta—immunoreactive)

Unsupervised patient stratification was performed by UnPaSt [31] (https://github.com/ozolotareva/unpast) v0.1.11, and applied to between-sample normalized and log2-transformed expression data with default parameters. UnPaSt standardizes each gene in the expression data matrix and searches for differentially expressed biclusters – submatrices consisting of genes concordantly over- or under-expressed in a subset of samples. Each such bicluster stratifies all samples into two subpopulations differentially expressing a certain gene set. We further refer to the smaller sample subset as the “bicluster” ($s_0$) and to the larger subgroup as the “background” ($s_1$ ); when $\left|s_0\right|=\left|s_1\right|$, the subset with higher expression on average is assigned to the “bicluster” group (Fig. 1A). In other words, UnPaSt identifies all gene sets in transcriptome data which define two well-separable sample groups, and cluster samples independently within each such gene set. Therefore, in contrast to conventional sample clusterings dividing samples into disjoint clusters based on all available genes (e.g. agglomerative hierarchical clustering with Ward’s linkage; Fig. 1B), UnPaSt reveals overlapping sample clusters and simultaneously extracts their specific genes, thus providing a more comprehensive and interpretable picture of data heterogeneity (Fig. 1C). Since UnPaSt is a probabilistic method, consensus biclusters were constructed from the results of ten independent runs using the approach described by Hartung et al., 2025 [31]. Only consensus biclusters supported by at least two runs were retained for downstream analysis.

To enable the detection of biclusters representing rare patients subgroups, the complete UKE dataset comprising 244 tumor samples was input to UnPaSt run with default parameters. Out of 234 biclusters, 177 biclusters defining two well-separable sample clusters (average signal-to-noise ratio (SRN) across all bicluster genes > 1.5), each including at least 10 samples from both treatment groups were selected for evaluation as predictive and prognostic biomarkers. SNR of a gene in a bicluster was calculated as the ratio of absolute difference of means $\mu_{s_0},\;\mu_{s_1}$ to the sum of standard deviations $\sigma_{s_0},\sigma_{s_1}$ computed for this gene expression across bicluster and background sample subsets $s_0$ and $s_1$:

$$SNR=\frac{\left|\mu_{s_0}-\mu_{s_1}\right|}{\sigma_{s_0}-\sigma_{s_1}}.$$

After the detection, only 181 samples selected for survival analysis were taken into account.

### Replication of biclusters discovered in the UKE data

To identify a bicluster in a validation dataset that resembles a previously discovered bicluster, samples from the validation dataset were divided into two clusters by k-means performed only for bicluster genes available. Further, individual genes from the new bicluster which poorly separated two sample clusters (SNR < 0.5) were removed. Only new biclusters with at least 2 genes and 10 samples were kept.

### Random survival forest

Random survival forest models [32] from *scikit-survival* [33] version 0.23.0 Python package were used to predict OS and PFS in treatment subgroups of the UKE cohort. Model inputs included patients’ age, tumor stage, surgical outcome, and expression levels of 23 biclusters, represented as binary variables.

For each treatment group from the UKE cohort, a random survival forest model was trained through a grid search approach with 5-fold cross-validation. Validation was performed within the corresponding treatment group of the DASL dataset. Individual feature importances were determined by averaging the decrease in model performance across 1000 permutations of the validation data, where the feature values were randomly shuffled [34].

### Statistical analysis

Time-to-event analysis and Kaplan–Meier curves were performed using *lifelines* Python package v0.27.7 [35]. Prognostic and predictive value of each biomarker candidate was assessed separately by fitting Cox Proportional Hazards models adjusted for donor’s age, operation result, and tumor FIGO. Stage and surgical outcome were modeled using indicator variables. Residual disease was categorized as no macroscopic tumor (reference), ≤ 1 cm, or > 1 cm. FIGO stage was categorized into stages IIIC, IV, or earlier stages (reference). Each biomarker candidate (molecular subtype or bicluster membership) was coded as a binary variable indicating presence versus absence of the feature.

- Survival ~ treatment*biomarker + age + stageIIIC + stageIV + residual_tumor ≤ 1  cm+ residual_tumor >1  cm.

To evaluate the effect of bevacizumab addition separately within biomarker-defined subgroups, additional Cox models were fitted within each subgroup:

- Survival~treatment + age + stageIIIC + stageIV + residual_tumor ≤1 cm+residual_tumor >1 cm.

Differential expression analysis was performed using *limma v*3.58.1 for the microarray-based DASL dataset and using *limma-voom* for the RNA-seq data [36]. Genes with at least 10 normalized counts in at least 10 samples were tested in the UKE and TCGA-OV datasets. Genes with adjusted *p*-values < 0.05 were considered to be differentially expressed.

For gene sets for overrepresentation (ORA) and gene set enrichment (GSEA) analyses, *gseapy* [37] python package v1.1.3 and gene set libraries *GO_Molecular_Function_2023*, *GO_Biological_Process_2023* [38, 39], Reactome_2022 [40], KEGG_2021_Human [41, 42], *WikiPathway_2023_Human* [43], *GTEx_Tissues_V8_2023* [44], *Descartes_Cell_Types_and_Tissue_2021* [45], *CellMarker_2024* [46], and *Tabula_Sapiens* [47] provided by *EnrichR* [48] were used. The subset of 20,376 genes expressed in the UKE cohort and successfully mapped to HGNC gene names was used as the background. Up- and down-regulated gene sets were tested for over-representation separately. Only overlaps with gene sets comprising 5 to 200 genes were tested, and only overlaps of at least two genes passing adjusted *p*-value cutoff of 0.05 (right-tailed Fisher’s exact test) were taken into account.

*P*-values were adjusted for multiple testing using Benjamini–Hochberg procedure [49].

### Signature-based public dataset search

To find datasets where genes from bicluster 84 were co-regulated we employed an unbiased dataset search approach described previously [50, 51]. First, we gathered a compendium of 44,259 public human gene expression matrices from NCBI GEO database [52], including microarray datasets available directly from NCBI GEO and RNA-seq gene count matrices obtained from ARCHS4 [53] and DEE2 [54] databases. For each of these datasets we ran Gene Set Co-regulation Analysis (GESECA) algorithm from the *fgsea* R package [55] using the 13 genes from the extended bicluster 84 signature as the input. The datasets then were sorted by GESECA *p*-value, so that the top hits would correspond to the lowest calculated *p*-values. Further, using the NLTK library we extracted terms associated with each dataset in the compendium. The hypergeometric test was then used to identify words overrepresented within the top 300 hits. The analysis pipeline is available as a web-service: https://alserglab.wustl.edu/coresh/ and described in detail by Sukhov et al., 2025 [51].

## Results

### The UKE ovarian cancer RNA-Seq cohort

**Fig. 2:**
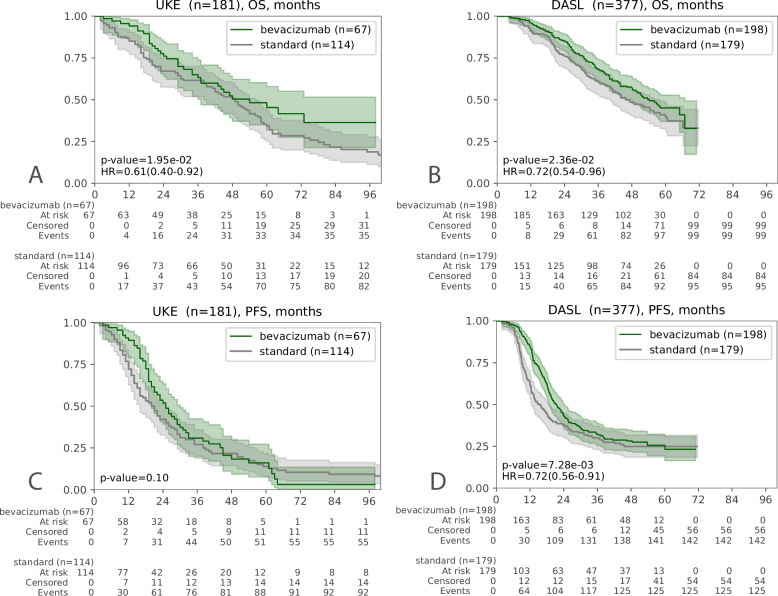
Kaplan–Meier plots for OS (**A**, **B**) and PFS (**C**, **D**) in bevacizumab and standard treatment groups of the UKE (**A**, **C**) and DASL (**B**, **D**) cohorts. X-axes show time from diagnosis in months. In the UKE cohort, bevacizumab addition to standard treatment significantly improved OS (HR = 0.61; *p*-value = 0.02), but the improvement was not statistically significant for PFS (HR = 0.75; *p*-value = 0.1). In the DASL cohort, the addition of bevacizumab to standard treatment significantly prolonged both OS (HR = 0.72; *p*-value = 0.02), and PFS (HR = 0.72; *p*-value = 0.007). The 95% confidence intervals (95% CI) for the survival curves are shown as shaded areas. For significant HRs 95% CI are reported in brackets

Most of the clinical characteristics of the UKE cohort summarized in Table 1 are similar to the characteristics of the DASL cohort previously published by Kommoss et al., 2017 [16]. In both cohorts, patients were treated with either platinum-based chemotherapy (standard) or with platinum-based chemotherapy and subsequent maintenance therapy with bevacizumab (bevacizumab). While the Kommoss study includes 189 and 170 patients in the bevacizumab and standard treatment groups, our cohort comprised 67 and 114 patients, who received bevacizumab and standard therapy respectively. The OS of these two patient subgroups is comparable in both cohorts (Fig. 2A and B). However, bevacizumab demonstrates a pronounced favorable impact on PFS in the DASL cohort, while in the UKE cohort prolongation of PSF under bevacizumab treatment is not statistically significant (Fig. 2C and D). This discrepancy may be attributed to the different distribution and number of patients in the treatment groups. In particular, the bevacizumab group in the UKE cohort included patients with more advanced tumors (FIGO stage III or IV) compared to the DASL cohort where 13.8% were at an early stage (FIGO stage I or II) [16].

#### Predictive value of individual genes

Since the expressions of certain genes were recently shown to be predictive for bevacizumab response in ovarian cancer [6–8, 11, 15], we evaluated predictive values of individual gene expressions in the UKE cohort. For this purpose, we employed the Cox Proportional Hazards models, which incorporated the patient’s age, tumor stage, and surgery outcome as covariates (expression of each gene was tested independently; see “Methods” for details). After the Benjamini–Hochberg procedure, no gene expressions were significantly predictive (adjusted *p*-value < 0.05) for OS or PFS in the UKE or DASL cohorts (Additional file 2: Fig. S2), including previously reported biomarker genes (Additional file 1: Table S3). Before adjustment for multiple testing, only *VEGFA* showed a nominal association with PFS in the DASL cohort (*p*-value = 0.04; HR = 1.32, 95% CI: 1.02–1.71) but not in the UKE cohort (*p*-value = 0.11; HR = 0.73, 95%CI: 0.50–1.07).

The lack of statistically significant single-gene biomarkers may be explained by the limited size of the UKE and DASL cohorts and high number of tested genes, and the associations between expressions and survival might be neglected after correction for multiple testing. Therefore, to mitigate the multiple testing problem, we performed supervised and unsupervised stratification of the UKE cohort based on the expression data, identified transcriptionally distinct sample sets and their specific biomarkers, and evaluated their predictive power in both cohorts.

#### Predictive value of known molecular subtypes

Molecular subtypes were determined using a random forest-based classifier implemented in *consensusOV* [26] R package (Additional file 1: Table S2; Methods). Out of 181 samples in the UKE cohort, 27 were classified as proliferative, 56 as mesenchymal, 48 as differentiated, and 50 as immunoreactive. Although patients who received bevacizumab in addition to standard treatment demonstrated improved OS, no molecular subtype was significantly predictive for OS or PFS in the UKE cohort (Table 2).

**Table 2:** Predictive value of molecular subtypes in the UKE cohort

	**Bevacizumab**	**Standard**	**OS**		**PFS**	
			HR (95%CI)	*p*-value	HR (95%CI)	*p*-value
Proliferative	13	14	0.73 (0.24–2.25)	0.59	1.58 (0.60–4.17)	0.35
Mesenchymal	23	33	1.14 (0.49–2.65)	0.75	0.53 (0.26–1.10)	0.09
Differentiated	7	41	0.56 (0.12–2.54)	0.45	1.11 (0.41–2.98)	0.83
Immunoreactive	24	26	1.46 (0.61–3.25)	0.39	1.35 (0.64–2.84)	0.43

Interaction *p*-values are not adjusted for multiple testing

Recently, *Kommoss *et al*.* suggested that molecular subtypes of ovarian cancer may demonstrate differential response to bevacizumab. They have shown that in the DASL cohort, patients with proliferative tumors may have improved PFS if bevacizumab is added to standard treatment [16]. Our analysis of the UKE cohort stratified by subtype also did not confirm this association, possibly due to limited cohort size (Additional file 2: Fig. S3). Other reasons for this discrepancy may be the smaller number and higher malignancy of patients in the UKE cohort or the difference in the approaches to the classification of patients into four molecular subtypes. Although molecular subtypes were shown to be prognostic for survival in many previous studies [24, 26–28, 30], in the UKE cohort, neither for patients under standard treatment only, nor for those who received bevacizumab, tumor subtypes demonstrated statistically significant association with OS or PFS in stratified analysis (Additional file 2: Fig. S4 and Additional file 2: Fig. S5).

#### Unsupervised patient stratification identifies reproducible expression signatures

Since no statistically significant associations between four ovarian cancer subtypes and bevacizumab response in the UKE cohort were found, we further searched for other expression-based predictors unrelated to known molecular subtypes. For that we employed UnPaSt [31], an unsupervised patient stratification method searching for differentially expressed biclusters in omics data. A differentially expressed bicluster in transcriptome data represents a submatrix stratifying all samples into two subpopulations with high or low expression of a certain gene set (see “Methods” and Fig. 1 for details). Some biclusters correspond to molecular pathways whose activity may define molecular subtypes or correlate with clinical characteristics of tumors, including drug response.

In total UnPaSt identified 234 differentially expressed biclusters consisting of 2–128 genes and 11–90 samples in the UKE dataset (Additional file 1: Table S4; Methods). However, due to the platform differences, many genes of these biclusters were absent in the DASL validation data. To focus the search on reproducible biomarker candidates, we first evaluated whether the genes comprising each bicluster discovered in the UKE data could also stratify samples into high- and low-expression groups in the DASL dataset. Consequently, 23 biclusters including at least two genes and presenting in both UKE and DASL cohorts were considered for further analysis.

### Random forest prioritizes predictive bicluster candidates

The 23 biclusters detectable in both UKE and DASL datasets were encoded as binary variables and used to train random survival forest models together with the patient age, operation result and FIGO stage. For each of the two treatment groups, an independent model was trained for OS and PFS on the UKE using five-fold cross-validation and validated on the DASL data. All four models achieved the performance of about 0.6 on the validation data, suggesting that they could only partially explain the observed survival. Feature importance analysis revealed that the strongest survival predictors were different for two treatment groups (Table 3). In the bevacizumab-treated group, patient age at diagnosis was the strongest predictor of both OS and PFS. In contrast, in the standard treatment group the importance of age was not high, and residual and tumor size > 1 cm and FIGO stage IV had the highest importance.

**Table 3:** The results of training random survival forest models to predict OS and PFS in treatment groups

	OS		PFS	
	**Bevacizumab**	**Standard**	**Bevacizumab**	**Standard**
Performance				
DASL (validation)	0.598	0.603	0.595	0.598
UKE (training)	0.749	0.687	0.646	0.677
Selected features				
Age at diagnosis	**0.040**	0.009	**0.032**	0.004
Bicluster 70	**0.021**	−0.001	**0.010**	0.001
Bicluster 84	**0.013**	0	−0.004	0
Bicluster 130	**0.010**	0	0.003	0.005
Bicluster 90	0.005	0	0	−0.001
Bicluster 109	0.001	0	0.002	0
Bicluster 41	0	−0.001	**0.016**	0.003
Bicluster 60	0	0.009	0	0.001
Bicluster 95	0	0.003	0.009	0
Bicluster 72	0	0	0	0.001
Bicluster 144	−0.004	0	−0.002	0.004
residual tumor > 1 cm	0.001	**0.045**	0.006	**0.026**
residual tumor ≤ 1 cm	0	0.009	**0.021**	**0.012**
FIGO IV	0	**0.014**	0	**0.039**
FIGO IIIC	0	0	0.004	0

The top two rows report model performance on the validation and training data, measured as the concordance index (c-index). The next rows provide the strongest predictors of OS and PFS specific to each treatment group selected based on their permutation-based importances. Permutation-based feature importance values exceeding 0.01 are shown in bold text font

Out of 23 selected biclusters, seven had positive importance for OS or PFS in the bevacizumab-treated group representing promising candidates for further investigation. Specifically, three biclusters were prioritized as potential determinants of differences in both OS and PFS (biclusters 70, 109, and 130), two for OS (biclusters 84 and 90), and two for PFS (biclusters 41 and 95). Kaplan–Meier plots for patients groups stratified by these bicluster candidates can be found in the Additional file 2: Fig. S6 and Additional file 2: Fig. S7. To explore their potential biological relevance, we performed ORA analysis of bicluster genes and searched for potential links with tumor vascularization and response to anti-VEGF therapy (Additional file 1: Table S5).

Bicluster 70, overrepresented with genes with endopeptidase activity (GO:0004175: *ADAM12, ADAMTS12, CORIN, CTSK, MMP11, MMP13, PLAU*; adj. *p*-value = 1.75e-04; OR = 12.4) and involved in extracellular matrix organization (GO:0030198; adj. *p*-value = 1.05e-03; OR = 16.9) had relatively high importance for both OS and PFS in the bevacizumab-treated group. Interestingly, in all three cohorts, stratification of samples by bicluster 70 signature strongly correlated with the pro- and anti-angiogenic classification of EOC proposed by Bentink et al., 2012 [56] (Additional file 2: Fig. S8). Consistent with their findings, tumors overexpressing these genes are predominantly classified as mesenchymal and immunoreactive. Bentink et al. also hypothesized that the presence of this angiogenic signature could inform decisions on bevacizumab administration. Paradoxically, our results suggest that, in both UKE and DASL cohorts, patients whose tumors overexpress the pro-angiogenic signature benefit less from the addition of bevacizumab in OS and shows decreased PFS compared to low-expression group (Additional file 2: Fig. S6E,F and Additional file 2: Fig. S7A,B). This finding suggests that these tumors may not only rely on VEGF to activate angiogenesis, but instead exploit alternative angiogenic pathways thus not being sensitive to bevacizumab. It is well known that there is a plethora of growth factors and pathways that tumor cells can exploit to induce angiogenesis and in several cancers these alternative angiogenic pathways have been described to be associated with reduced tumor susceptibility to anti-VEGF agents [57].

The bicluster 130 signature (Additional file 2: Fig. S9A-C) is enriched with genes specific to the adipose tissue (*ADIPOQ, CIDEA, FABP4*; adj. *p*-value = 6.72e-05; OR = 92.7) and related to lipid metabolism. Most of the tumors overexpressing this signature are classified as mesenchymal and from about one third to more than a half of all mesenchymal tumors overexpress these genes. In both cohorts, patients with low bicluster 130 expression who received bevacizumab showed improved PFS and markedly better OS than bevacizumab-treated patients whose tumors overexpressed this signature and patients who did not receive bevacizumab (Additional file 2: Fig. S6C,D and Additional file 2: Fig. S7K,L). Adiposity-related features have previously been associated with reduced benefit from bevacizumab in ovarian cancer [58, 59] and in other cancer types [60, 61], although findings have not always been consistent across studies [62]. One possible explanation of this association is that adipose tissue produces angiogenesis-regulating factors, including VEGF [63]. The discrepancies across independent studies may reflect differences in clinical characteristics of patient cohorts and in the methods used to quantify adiposity features. In addition to adipose tissue gene expression, bicluster 130 also showed increased expression of markers of immune cells (e.g. *CCR7, CD79B, CST7, EOMES, FCRLA, IL1RL1, ITK*, and *TNFRSF13B*) which may also contribute to angiogenesis regulation [64]. *CD36*, also upregulated in these tumors, represents a known negative regulator of angiogenesis and its overexpression may reflect the pre-existing inhibition of the VEGF signal [65, 66] making the addition of bevacizumab inefficient.

Bicluster 90 consists of *NR4A1* and *NR4A3*, which are involved in VEGF signaling [67–69] (*VEGFA-VEGFR2* Signaling WP3888; adj. *p*value = 1.12e-02; OR = 47.2). In both cohorts, we observe improved OS in patients with low expression of *NR4A1* and *NR4A3* who receive bevacizumab, compared with those who have high expression of these genes. In this subgroup, survival is comparable to that of patients who do not receive bevacizumab, regardless of *NR4A1* and *NR4A3* expression levels (Additional file 2: Fig. S6G,H). We hypothesize that tumors with high *NR4A1* and *NR4A3* expression support endothelial development independently of VEGF*,* or already possess an established vascular network. In contrast, tumors with low *NR4A1* and *NR4A3* expression may still be actively developing vasculature, making this process more susceptible to inhibition by anti-VEGF therapy.

All five genes comprising bicluster 41 (*FOXD1, PAX6, ZIC2, ZIC3, ZIC5*) are annotated as cis-regulatory region sequence-specific DNA binding (GO:0000987; adj.*p*-value = 2.17e-6), significantly overlap with the Mesodermal Commitment Pathway (WP2857; adj. *p*-value = 1.38e-7; OR = 595.1) and genes overexpressed in the central nervous system (Brain-Cerebellum, female, GTEx v8; adj.*p*-value = 3.86e-9; OR = 1040.7). Given that mesodermal lineages give rise to multiple vascular cell types, the activity of these genes may be linked to angiogenic processes. In both cohorts, patients with high expression of this signature exhibit a trend toward improved PFS across both treatment groups (Additional file 2: Fig. S7E,F); however, its predictive and prognostic value requires validation in larger cohorts.

Bicluster 95 consisted of two HOX genes, *HOXB8* and *HOXB9*, both annotated with the GO terms *double-stranded DNA binding* (GO:0003690; adj. *p*-value = 0.01; OR = 36.6) and *regulation of DNA-templated transcription* (GO:0006355; adj. *p*-value = 0.04; OR = 10.8). Notably, *HOXB9* expression has been previously linked to response to bevacizumab in colorectal cancer [70]. Specifically, under bevacizumab treatment patients with low *HOXB9* expression in tumors showed prolonged PFS compared with those with high expression, and silencing of *HOXB9* restored bevacizumab sensitivity in mice models [70]. Our observations in EOC, however, suggest the opposite trend, indicating that patients with elevated expression of these two genes may demonstrate longer PFS (Additional file 2: Fig. S7G,H).

For biclusters 84 and 109 consisting of five and nine genes respectively, no significant overlaps with any tested gene sets were found.

### Statistical evaluation of prioritized biclusters

Further, the predictive value of each bicluster candidate prioritized by random survival forest was evaluated for statistical significance using a Cox model adjusted for patient’s age, tumor stage, and surgery outcome. Bicluster 84 represented the most promising predictive biomarker candidate for the OS under bevacizumab treatment compared to the standard treatment in both cohorts (Table 4).

**Table 4:** Interaction between treatment and bicluster candidates predictive for OS and PFS selected by random survival forests

	UKE (*n* = 181)			DASL (*n* = 377)		
	***p*-value**	**adj. *p*-value**	**HR (CI 95%)**	***p*-value**	**adj. *p*-value**	**HR (CI 95%)**
OS						
Bicluster 70	0.09	0.23	0.42 (0.16–1.14)	0.51	0.80	1.21 (0.68–2.16)
Bicluster 84	0.07	0.23	2.1 (0.94–4.66)	0.01	0.07	2.06 (1.16–3.66)
Bicluster 130	0.29	0.36	1.59 (0.68–3.74)	0.14	0.34	1.77 (0.84–3.72)
Bicluster 90	0.18	0.31	1.75 (0.77–4.01)	0.92	0.92	1.03 (0.58–1.82)
Bicluster 109	0.53	0.53	1.31 (0.56–3.02)	0.64	0.80	1.17 (0.61–2.23)
PFS						
Bicluster 70	0.88	0.99	1.06 (0.52–2.15)	0.95	0.97	1.02 (0.62–1.66)
Bicluster 130	0.79	0.99	1.10 (0.54–2.23)	0.53	0.91	0.81 (0.41–1.57)
Bicluster 109	0.58	0.99	1.22 (0.60–2.52)	0.97	0.97	1.01 (0.56–1.81)
Bicluster 41	0.99	0.99	1.0 (0.49–2.04)	0.54	0.91	1.21 (0.65–2.25)
Bicluster 95	0.95	0.99	1.02 (0.49–2.13)	0.02	0.08	0.54 (0.33–0.9)

Each bicluster candidate was tested independently, the resulting *p*-values were adjusted following the Benjamini–Hochberg procedure

**Fig. 3:**
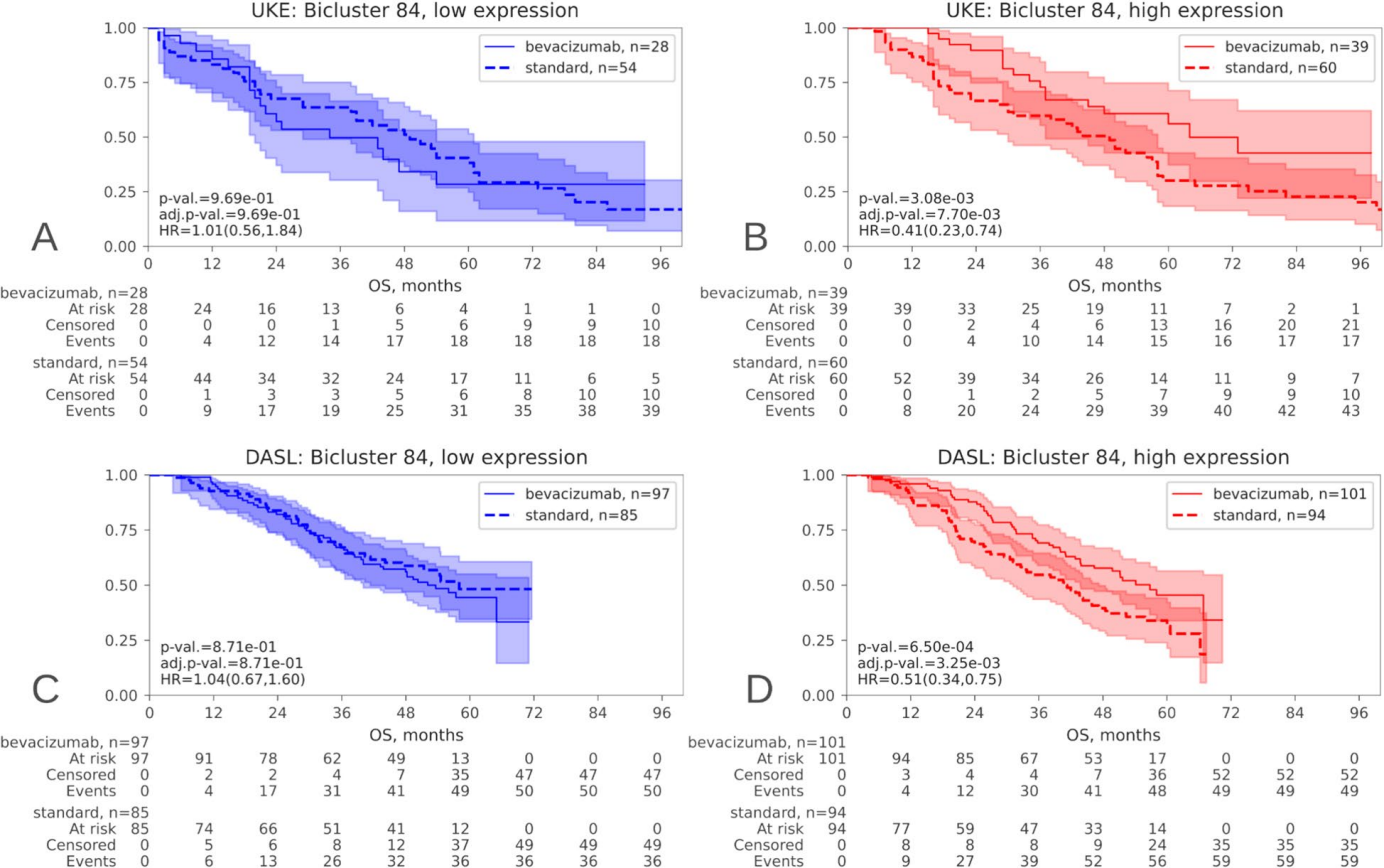
Kaplan–Meier plots for OS in the bevacizumab (solid lines) and standard (dashed lines) treatment groups of the UKE (**A**, **B**) and DASL (**C**, **D**) cohorts stratified by expression of bicluster 84 genes. Hazard ratios (HR) estimate the ratios of death risks between the bevacizumab-treated group and the standard treatment group. In both cohorts, patients whose tumors over-expressed (red) bicluster 84 genes demonstrated longer OS when bevacizumab was added to standard chemotherapy. The OS of patients with tumors under-expressing bicluster 84 genes (blue) was not significantly different between treatment groups. Since each of five bicluster candidates were tested independently, the resulting *p*-values were adjusted following the Benjamini–Hochberg procedure. The shaded areas around curves show the 95% confidence intervals

In the DASL cohort, bicluster 84 signature significantly interacted with treatment (*p*-value = 1.36e-02) and demonstrated the same tendency in the UKE cohort, although it did not pass the commonly used threshold for statistical significance (*p*-value = 6.92e-02). In both cohorts, patients with overexpression of bicluster 84 genes in the tumor tissue benefited from the addition of bevacizumab to standard treatment, whereas bevacizumab treatment of patients with low expression of bicluster 84 did not provide any benefits compared to standard platinum-based therapy (Fig. 3 and Additional file 2: Fig. S6A,B).

In the subset of the UKE cohort including 99 patients whose tumors over-expressed bicluster 84 genes, bevacizumab-treated group demonstrated a prolonged OS, compared to those who only received standard treatment (*p*-value = 3.08e-03; adj.*p*-value = 7.70e-03; HR = 0.41, 95% CI: 0.23–0.74). In the DASL cohort, bevacizumab addition caused the same effect on the OS of patients who over-expressed this signature (*p*-value = 6.50e-04; adj.*p*-value = 3.25e-03; HR = 0.51, 95% CI: 0.34–0.75). In both cohorts, no significant differences in the OS between treatment groups were observed among the patients whose tumors under-expressed the bicluster 84 genes (*p*-value = 0.97; HR = 1.01, 95% CI: 0.56–1.84, and *p*-value = 0.87; HR = 1.04, 95% CI: 0.67–1.60, in the UKE and DASL datasets respectively).

#### Bicluster 84 signature replicates across datasets and platforms

Bicluster 84 consisted of five genes in the UKE cohort, of which two, the solute carrier organic anion transporter family member 6A1 (*SLCO6A1*) and the parathyroid hormone 2 receptor (*PTH2R*), were also measured in the DASL. Three other genes encoding a pseudogene (*ENSG00000249721*) and long non-coding RNAs (*LINC00491, LINC01456*), were absent from the DASL data.

**Fig. 4:**
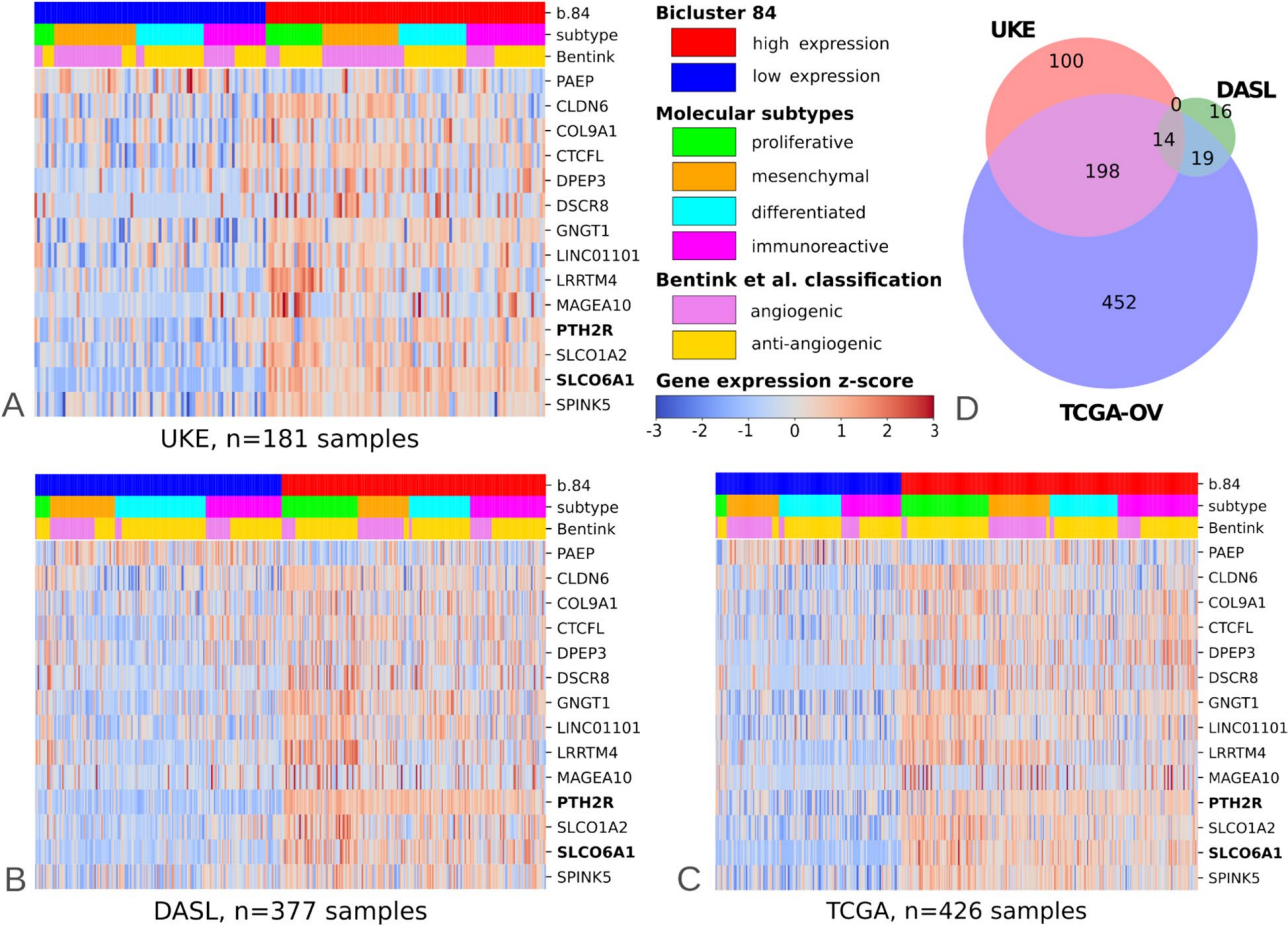
Normalized expressions of the 14 genes significantly differentially expressed by bicluster 84 signature (adj.*p*-value < 0.05, |log2FC|> 1) in the UKE (**A**), DASL (**B**), and TCGA-OV (**C**) cohorts. All genes except PAEP are down-regulated in the patient subgroup included in bicluster 84 (blue bar on the top of the heatmap). Gene included in bicluster 84 discovered in the UKE dataset are highlighted with bold text font. Red and blue bars on the top of the heatmaps highlight samples over- and under-expressing bicluster 84 genes. Sample classification with known molecular subtypes determined by *consensusOV* (green—proliferative, orange—mesenchymal, cyan—differentiated, magenta—immunoreactive) is shown with the colorbar on the top of the heatmap. ENSEMBL gene ids were mapped to HGNC symbols.** D** The overlaps of significantly differentially expressed gene sets for bicluster 84 signature in the UKE, DASL, and TCGA-OV datasets

Because UnPaSt is a probabilistic method which constructs biclusters from genes defining sharp sample clusters, many genes satisfying commonly used threshold of 0.05 for statistically significant differential expression between bicluster and background sample groups but having low signal-to-noise ratio (SNR), may be not included in biclusters. Therefore, to better understand biological roles of bicluster 84 signature and to connect it with bevacizumab response, we expanded it by extracting all genes statistically significantly differentially expressed between patient subgroups defined by this bicluster. To reduce the number of false findings, we focused on genes which were significantly differentially expressed in both UKE and DASL cohorts. Out of 312 and 49 genes significantly differentially expressed (|log2(FC)|> 1, adj.*p*-value < 0.05) between patient groups stratified by bicluster 84 in the UKE and DASL cohorts respectively, 14 genes were shared (Fig. 4A, B Additional file 1: Table S6). This overlap of two gene sets is stronger than expected by chance (one-sided Fisher’s exact test *p*-value = 1.80e-19, 15,583 genes present in both cohorts were taken into account).

Additionally, we validated the presence of bicluster 84 signature in another publicly available RNA-seq based ovarian cancer cohort which included samples from patients who did not receive bevacizumab, TCGA-OV (Fig. 4C). The list of 683 genes differentially expressed in the TCGA-OV demonstrated even stronger overlap with the genes from the UKE dataset (212 shared genes; *p*-value = 6.30e-269; 22,491 genes presenting in both UKE and TCGA-OV were taken into account, Fig. 4D). The better concordance between the expression signatures in UKE and TCGA-OV compared to DASL, may be attributed to the use of microarrays in the latter, whereas in the UKE and TCGA-OV gene expression was profiled by RNA-seq.

Interestingly, in all three cohorts, tumor subsets defined by bicluster 84 did not exclusively match any of four established molecular subtypes of EOC (Fig. 4A-C). Tumours overexpressing this signature represented all four molecular subtypes in roughly equal proportions, although the majority of the samples classified as proliferative fall into the subgroup overexpressing bicluster 84 genes. These observations suggest that bicluster 84 may correspond to biological processes unrelated to molecular subtypes and define an additional classification of tumours. To better understand its biological role and find possible connection with the response to bevacizumab, we reviewed the fourteen genes included in this signature in all three datasets, and compared it with known pathways, functionally similar and tissue specific gene sets.

### Characterization of extended bicluster 84 signature

Among the fourteen genes significantly differentially expressed in tumor subgroups defined by bicluster 84, only *PTH2R* has been previously directly linked with angiogenesis. *PTH2R* is a selective parathyroid hormone receptor normally expressed at high levels in the brain and central nervous system and described as a key mediator of nociception and maternal behavior [71, 72]. Three endogenous ligands have been described for *PTH* receptors: tuberoinfundibular peptide of 39 residues (*TIP39*), *PTH*, and *PTH*-related peptide (*PTHrP*) [73]. *PTH2R* is also involved in the wound healing process, where ligand *TIP39*-mediated signaling induces the expression of decorin and the maturation of the extracellular matrix [74]. Also, *PTH* and *PTHrP* have been associated with angiogenesis activation [75, 76]. There are few reports about the role of *PTH2R* in cancer, and interestingly among them a recent work identified a significant up-regulation of *PTH2R* in ovarian cancer cells and could further demonstrate its association with tumor cell proliferation, invasion and migration [77].

The *SLCO6A1* gene encodes a protein called *OATP6A1*, which belongs to the solute carrier organic anion transporter family and has been identified as a cancer-testis antigen in lung tumor cell lines [78] and tumors of the lung, bladder, esophagus, and brain. Also *LINC00491* has been implicated in various cancer types such as pancreatic cancer and lung cancer, promoting cell growth and metastasis [79, 80].

Besides *PTH2R* and *SLCO6A1*, twelve other genes were differentially expressed in tumors with overexpression of bicluster 84 signature with an absolute log2-fold-change above 1 in both datasets (Additional file 1: Table S6). Same as *SLCO6A1*, three of them were cancer germline genes: *DPEP3, MAGEA10*, and *CTCFL*. The latest, also known as BORIS, encodes a master transcription factor that maintains cancer stemness by regulating several oncogenes and also other CTAs in different cancer types [81–85]. *CTCFL* is strongly expressed and frequently amplified in ovarian and other cancers [86], where it regulates *VEGF-A* expression alongside other factors [87]. Recently, it has been shown to maintain treatment-resistant phenotype of cancer cells [88]. Two extracellular matrix-related genes *COL9A1* and *CLDN6* were previously shown to be overexpressed in the C5 subtype of EOC defined by Tothil et al. [89]. Also, *COL9A1*, which encodes collagen IX, has been previously shown to exert an anti-angiogenic function, by increasing the levels of antiangiogenic TSP-1 and matrilin-1 and decreasing the levels of proangiogenic active MMP-9 [90]. However, this association was not consistently recapitulated in our data. Only a modest increase in *MATN1* was observed in *COL9A1*-high vs *COL9A1*-low patient subgroups stratified by median *COL9A1* in the DASL cohort (adj. *p*-value = 2.89e-3; Mann–Whitney test; difference of standardized expression medians = 0.46), but not in the UKE cohort (adj. *p*-value = 0.42). SPINK5 is a serine protease inhibitor, contributing to the anti-inflammatory and/or antimicrobial protection of mucous epithelia and described as a tumor suppressor [91]. In a renal cell carcinoma cohort treated with atezolizumab plus bevacizumab, responders expressed lower levels of SPINK5 than non-responders [92], suggesting that the relationship between SPINK5 expression and treatment response may be context-dependent. *GNGT1, LRRTM4,* the long non-coding RNAs *LINC01101* and DSCR8 have been previously discussed in the context of cancer, but to our knowledge, no association with angiogenesis has been reported so far.

The only gene demonstrating an opposite expression pattern to the other 13 genes of this signature, *PAEP*, encodes a 28-kDa glycoprotein glycodelin that participates in tumor angiogenesis by promoting endothelial cell proliferation, migration and tube formation through modulation of *VEGF-A* expression [93]. Glycodelin has been previously shown to be increased in plasma of patients with gynaecological malignancies, including ovarian cancer where it was correlated with poor prognosis at advanced stages [94]. Why the patients who respond to anti-angiogenic therapy have low levels of the angiogenic factor glycodelin *PAEP* remains to be clarified.

Although we validated the presence of this expression signature in three independently generated datasets, both over-representation and GSEA analyses provided limited insight into its biological role. Gene set over-representation analysis (ORA) of the 13 upregulated genes revealed small but significant overlaps with pathways related to testis, confirming the aforementioned presence of cancer/testis antigens (*CTCFL, DPEP3, MAGEA10*; adj. *p*-value = 2.51e-4; OR = 125.9), transmembrane transport activity (GO:0015347; *SLCO1A2* and SLCO6A1; adj. *p*-value = 7.75e-4; OR = 314.3), anti-inflammatory response favoring leishmania infection (R-HSA-9662851; *DPEP3, GNGT1, PTH2R*; adj. *p*value = 1.48e-2; OR = 39.6), and external encapsulating structure organization (GO:0045229; *COL9A1* and *SPINK5*; adj. *p*-value = 3.95e-2; OR = 29.0; Additional file 1: Table S7A). ORA analysis of a broader set of 209 genes significantly upregulated in both RNA-seq-based datasets (TCGA-OV and UKE) revealed over-representation of genes specific to neurons (*CDH6, CNTN5, CNTNAP5, FXYD7, GABRA3, OLFM1, PHOX2A, PTPRR, SLC12A1, SLC44A5, TMEM132B, TUBB2B*; adj. *p*-value = 6.37e-7, OR = 10.6) and kidney (*ADAMTS20, CDH6, CLEC18C, KIF1A, LRP2, PCDH15, SLC12A1, TRIM71*; adj. *p*-value = 5.99e-6, OR = 14.4) in addition to testis-specific genes (adj. *p*-value = 6.30e-06, OR = 28.9; *CTCFL, DPEP3, MAGEA10, MAGEA11, MAGEA4, MAGEC1, STK31*; Additional file 1: Table S7B). GSEA analysis confirmed the enrichment of genes specific to testis tissue among the genes differentially expressed in tumors stratified by bicluster 84 in the UKE dataset, but no associations passed the adjusted *p*-value cutoff of 0.05 for the DASL and TCGA-OV datasets (Additional file 1: Table S7C).

Since both ORA and GSEA analyses showed that known gene sets are not sufficient to explain bicluster 84 signature, we employed CORESH (see “Methods”) to search the Gene Expression Omnibus (GEO) database for public datasets where these genes are co-regulated. Among 44,259 human-derived datasets, CORESH identified 128 where the bicluster 84 genes were co-regulated stronger than expected by chance (adj. *p*-value < 0.05; Additional file 1: Table S8A). Text mining analysis of the titles and descriptions revealed overrepresentation of the word “pluripotent” (adj. *p*-value = 2.71e-23; fold-enrichment = 4.52) and other terms associated with stemness among the top-ranked datasets (Additional file 1: Table S8B). We hypothesize that this expression signature may be driven by *CTCFL* (*BORIS*) [81, 82], which has been shown to maintain stemness and treatment-resistant phenotype of cancer cells [88]. *CTCFL* is frequently amplified in ovarian and other cancers [86] and recently has been shown to regulate *VEGF-A* expression [87]. Previous studies have shown that *CTCFL* can regulate the expression of pluripotency- and stemness-associated markers [82, 95]. In the UKE dataset, embryonic stem cell markers *SOX2, POU5F1,* and *MYC* were not differentially expressed in patients stratified by bicluster 84. However, we observed significant overexpression of the cancer stem cell markers CD24 (adj. *p*-value = 0.02; logFC = 0.79) and *EPCAM* (adj. *p*-value = 0.02; logFC = 0.61) in the *CTCFL*-high subgroup. Consistently, *EPCAM* was also overexpressed in the *CTCFL-*high subgroup of the DASL cohort (adj. *p*-value = 0.01; logFC = 0.2), CD24 was overexpressed in the TCGA-OV *CTCFL*-high subgroup (adj. *p*-value = 0.04; logFC = 0.31). Besides *CTCFL*, the 14-gene signature also includes *CLDN6*, a tight-junction protein, that is largely absent from adult tissues and normally expressed during early embryonic development in pluripotent stem cells [96], and also elevated in some tumors [97]. Additionally, *MAGEA10* overexpression has been associated with poor differentiation and activation of germline programs [98], which may contribute to stemness-like phenotypes.

### Impact of platform on reproducibility of expression signatures

While evaluating the biclusters in the TCGA-OV data, we noticed that in general, biclusters discovered in the UKE dataset replicated in this RNA-seq-based cohort much better than in the DASL (Additional file 2: Fig. S10). All 23 biclusters replicated in the DASL data were also found in the TCGA-OV, and many biclusters that failed to replicate in the DASL were successfully identified in the TCGA-OV. Additionally, biclusters replicated in the TCGA-OV retained more genes than their counterparts in the DASL. This higher concordance between UKE and TCGA-OV is likely due to both cohorts being profiled using RNA-seq technology, whereas DASL is microarray-based and lacks or imprecisely measures some genes. One of the most illustrative examples of this limitation is bicluster 129, consisting of 128 genes in the UKE data, the majority of which encode immunoglobulin or T-cell receptor chains. In the DASL cohort, only 19 out of 129 genes were measured and only 6 were included in the bicluster. In contrast, the majority of the 129 genes were present in the TCGA-OV, and 80 were included in this bicluster. Interestingly, in all three cohorts, only about half of samples included in this bicluster were classified as immunoreactive (Additional file 2: Fig. S11). Overall, 97 biclusters discovered in the UKE data and replicated in the TCGA-OV did not match any of four molecular subtypes (adjusted Rand index < 0.25) were identified. Such reproducible expression patterns may have biological or clinical significance and might be of interest for future research. However, because the patients in the TCGA-OV cohort only received chemotherapy [24] we cannot draw any conclusion about their association with survival under bevacizumab treatment.

To further assess whether prioritized signatures derived from transcriptome data are also reflected at the protein level, we analyzed publicly available MS-based ovarian cancer proteomic datasets from Hu et al. [99] and McDermott et al. [100]. Coverage of the seven prioritized biclusters in these data was limited, with only 12 and 31 proteins measured in the Hu et al. and McDermott et al. datasets, corresponding to 11% and 29% of the genes in these biclusters, respectively. The *CTCFL* protein expression was measured in both datasets. Its abundance fell within the intermediate range relative to all measured proteins and varied substantially across tumors (Additional file 2: Fig. S12A-D). In the McDermott et al. cohort, *CTCFL* and *CLDN6* showed coordinated expression patterns consistent with the 14-gene signature identified in RNA-seq data. Several genes from bicluster 70 also showed heterogeneous protein-level expression in both datasets, and *ADIPOQ* and *FABP4* from bicluster 130 also formed a bicluster in the McDermott et al. dataset (Additional file 2: Fig. S12E,F). Overall, these results suggest partial replication of the signatures at proteomic level, but their further investigation and correlation with survival is required.

## Discussion

The anti-angiogenic therapy is one of standard maintenance treatment options for advanced ovarian cancer, however, reliable biomarkers to guide the use of bevacizumab are still lacking. To address this urgent need for bevacizumab response biomarkers, we explored a novel dataset including expression profiles and clinical data of EOC patients treated at Universitätsklinikum Hamburg-Eppendorf (UKE). Although our analysis of the UKE dataset did not confirm associations between molecular subtypes of EOC and bevacizumab response in terms of OS or PFS, biclustering analysis revealed multiple expression signatures which replicated in independently generated EOC expression datasets (DASL, TCGA-OV) and were unrelated to known molecular subtypes. One of them, bicluster 84, was prioritized by random survival forest as a predictive biomarker candidate associated with OS under bevacizumab treatment. Although its interaction with treatment in Cox regression model adjusted for patient age, tumor stage and surgery outcome, did not pass the commonly used threshold for statistical significance (UKE: p-val. = 6.92e-02, adj. p-val. = 0.23; DASL: p-val. = 1.36e-02, adj.p-val. = 0.07), probably due to lack of power, stratified analysis results suggest its potential prognostic value. For patients whose tumors overexpressed this signature, bevacizumab addition to standard treatment significantly prolonged OS in both UKE (HR = 0.41, 95% CI: 0.23–0.74; *p*-value = 3.08e-03; adj.*p*-value = 7.70e-03) and DASL (HR = 0.51, 95% CI: 0.34–0.75; *p*-value = 6.50e-04; adj.*p*-value = 3.25e-03) cohorts. In the rest of patients, no significant differences in OS explained by bevacizumab addition were observed. Further, because traditional functional over-representation approaches relying on known gene sets aided limited support in finding biological interpretation of this expression signature, we performed mining of public expression data from NCBI GEO and identified potential connection of this signature with the acquisition of stemness-like properties. We hypothesized that it may be driven by *CTCFL* (*BORIS*), a key regulator of cancer stemness [81, 82].

The acquisition of stemness properties by cancer cells promotes their growth, invasiveness, and increases their drug resistance. A link between cancer cell stemness and angiogenesis activation, e.g. through secretion of angiogenic factors such as *VEGF-A*, has been described for cancer stem cells in general and for ovarian cancer cells in particular [101–103]. Therefore, an overexpression of this signature in the tumor at the beginning of treatment may be associated with the effectiveness of anti-angiogenic therapy. Furthermore, Krishnapriya et al. described the contribution of cancer stem cells in high-grade serous ovarian cancer to the formation of blood and lymphatic vessels [104]. Spheroids derived from ascites-derived cells cultured in stem cell medium showed high expression of endothelial, pericyte and lymphatic endothelial markers and were able to form endothelial and lymphatic tube-like structures in vitro and in vivo. Notably, bevacizumab inhibited the differentiation of spheroids into endothelial cells in vitro. These results suggest that cancer stem cells may themselves contribute to angiogenesis in EOC and that this process can be inhibited by anti-angiogenic treatment with bevacizumab [104]. Taken together, previous evidence and our new findings underscore the need for further studies to define the biological role of the bicluster 84 signature and to confirm a central causal role for *CTCFL*. If validated, this signature may not only help guide bevacizumab administration, but also inform the selection of *CTCFL*-targeted therapies [105, 106], and complement the existing molecular classification of EOC.

It is important to note that our models only partially explained the observed differences in survival between treatment groups. Our analysis indicates that in both the discovery and validation cohorts, different molecular and clinical variables are more relevant for prediction of OS than PFS. OS is considered the gold standard endpoint in cancer clinical trials because it can be accurately measured from the documented dates of death, and has a low risk of reporting bias [107]. In contrast, PFS, though also an objective primary endpoint, may be more susceptible to observational bias. Particularly in ovarian cancer, the limitations of imaging systems, but also of the main biomarker *CA125*, and the different timing of assessment contribute to the complexity of using PFS as a surrogate endpoint for OS in ovarian cancer and highlight the need for careful consideration when interpreting results based on PFS alone.

Furthermore, given the high molecular heterogeneity of EOC and its unexplored diversity beyond the four molecular subtypes, it is possible that bevacizumab efficacy may be determined by a combination of multiple expression signatures. However, due to the limited size of the datasets available, in this work we explored only individual effects of the biclusters and have not statistically tested the effects of their combinations. Therefore, to comprehend this heterogeneity and to develop a stronger predictor of bevacizumab response, replication of this study with larger cohorts is required.

Besides the limited cohort size, another challenge in our analysis was the cross-platform validation of expression signatures discovered in RNA-seq data. Since some genes from the discovered biclusters were not represented in the dataset generated using microarray technology, the validation step may have excluded genes with potential biological or clinical relevance. As a result, the original bicluster could not always be fully reconstructed, which might have complicated its evaluation by weakening the robustness of sample clustering. This highlights the need for an independent validation cohort of EOC patients treated with bevacizumab whose tumor expressions would be profiled using RNA-seq technology. In addition, our analysis was primarily focused on transcript level measurements; further research on bevacizumab response biomarkers would benefit from future studies incorporating large proteomic datasets in matched treatment settings. Finally, in this work, we focused on identifying tumor-derived biomarkers, and the search of circulating biomarkers of bevacizumab response will be an important next step in future research.

## Conclusions

In this work, we applied unsupervised and supervised machine learning methods to investigate the transcriptional heterogeneity of EOC beyond established molecular subtypes, and identified several expression signatures, reproducible across datasets and may be predictive of benefit from bevacizumab. Statistical analysis prioritized previously undescribed signature potentially predictive for benefit from bevacizumab and enriched with cancer/testis antigens. We hypothesized that overexpression of this signature may be associated with the acquisition of stemness-like properties by cancer cells through *CTCFL* activation. Given the high heterogeneity of EOC and that this novel signature only partially explains variation in survival outcomes, larger RNA-seq datasets are required to uncover additional biomarkers of bevacizumab response and to further improve predictive models.

## Supplementary Information

Additional file 1: Table S1. The raw read counts and sample metadata for the UKE cohort. Table S2. Molecular subtypes of ovarian cancer. Table S3. Predictive value of genes previously reported as bevacizumab response biomarker candidates for OS and PFS. Table S4. 234 biclusters identified in the UKE dataset (*n* = 244). Table S5. Composition of biclusters prioritized by RSF. Table S6. Genes significantly differentially expressed in samples stratified by bicluster 84. Table S7. ORA and GSEA results. Table S8. The results of public expression data mining.

Additional file 2: Supplementary Fig. S1-S12 with legends.

## Data Availability

The raw read counts and sample metadata generated during this study are available in Additional file 1: Table S1 of this article. Raw sequencing data from the UKE cohort are not publicly available due to patient consent restrictions. Data may be shared for scientific purposes upon reasonable request to the corresponding author, Leticia Oliveira-Ferrer, subject to institutional approval. Expression data for the DASL cohort is available in NCBI GEO (accession GSE140082 [108]), with sample metadata provided by Prof. Stefan Kommoss. The TCGA-OV dataset is publicly accessible via Xena (https://xenabrowser.net/datapages/?cohort=TCGA%20Pan-Cancer%20(PANCAN)) [109]. The code used to perform data analysis described in this article is available at https://github.com/ozolotareva/bevacizumab_ovca_signature [110] and is also deposited in Zenodo at https://zenodo.org/records/21264218 [111].

## Abbreviations

BH: Benjamini–Hochberg procedure
DASL: CDNA-mediated annealing, selection, extension, and ligation array
EOC: Epithelial ovarian cancer
GSEA: Gene set enrichment analysis
GOG-0218: Gynecologic Oncology Group study 218
ICON7: International Collaboration on Ovarian Neoplasms
ORA: Gene set over-representation analysis
OS: Overall survival
PFS: Progression-free survival
RNA-seq: Sequencing of ribonucleic acids
SNR: Signal-to-noise ratio
TCGA: The Cancer Genome Atlas
TCGA-OV: Ovarian cancer dataset from The Cancer Genome Atlas
UKE: Universitätsklinikum Hamburg-Eppendorf - University Medical Center Hamburg-Eppendorf
95% CI: 95% Confidence intervals
