# Supplementary material for: Transcriptome signatures for the identification of bevacizumab responders in ovarian cancer
Source: Genome Med. 2026 Aug 5;18:121. doi: 10.1186/s13073-026-01727-6 (PMC13455466; doi:10.1186/s13073-026-01727-6)
Supplement: Supplementary file 1 [file 13073_2026_1727_MOESM2_ESM.pdf]

Supplementary Figures

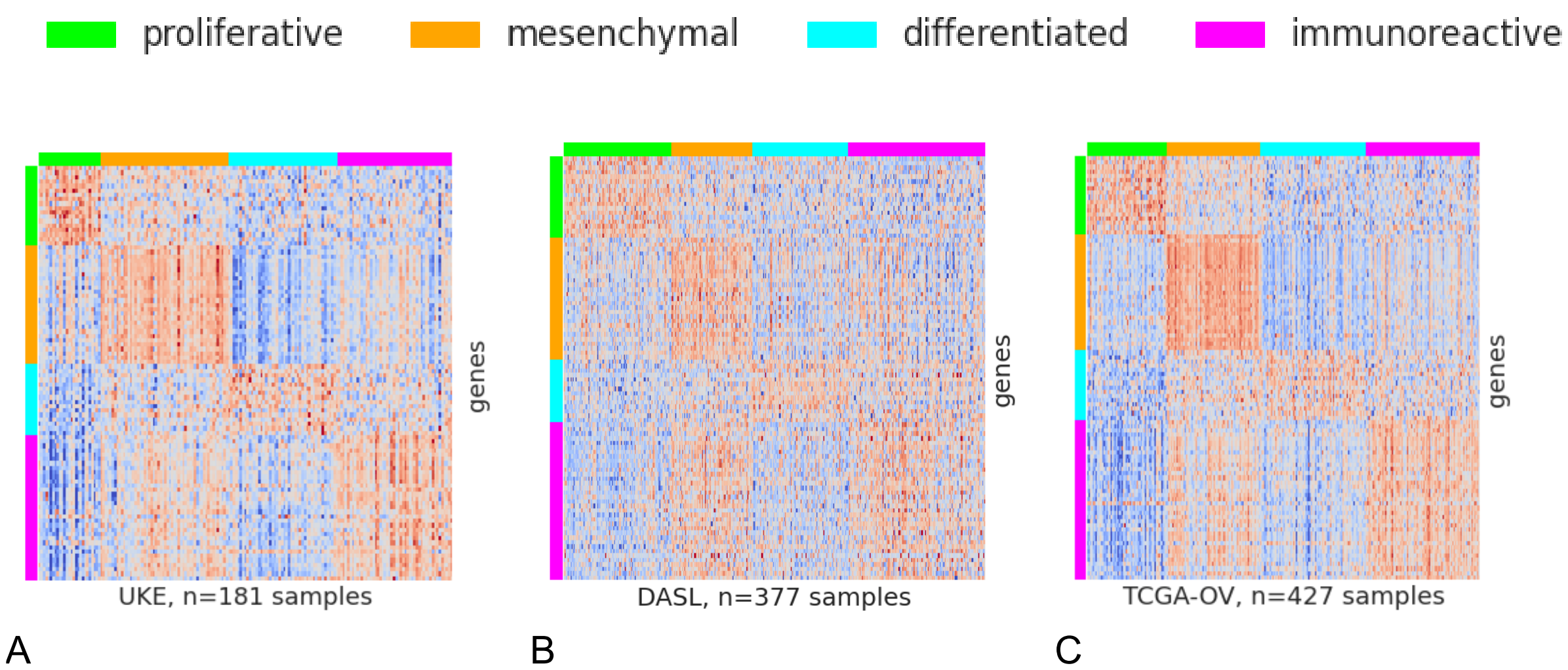

Fig. S1. Molecular subtypes of ovarian cancer predicted in the UKE (**A**), DASL (**B**), and TCGA-OV (**C**) cohorts assigned by the *consensusOV* classifier[2]. Normalized and standardized expressions of 98 genes with subtype-specific expressions manually curated from Verhaak et al.[3] and Talhouk et al.[4] are shown. The list of subtype-specific biomarkers obtained by combination of biomarkers used for ovarian cancer subtyping used by Talhouk et al. and Verhaak et al. and measured in the UKE, DASL and TCGA-OV datasets. The following 16 genes were excluded due to low concordance with molecular subtypes assigned in TCGA-OV dataset: ABCC3, TMEM45A, PDZK1IP1, CD74, SCGB2A2, COPS3, MFAP2, OLFML3, RAD51AP1, BLMH, C1ORF116, C5ORF13, ECGF1, HRBL, TMEM24, RARRES3.

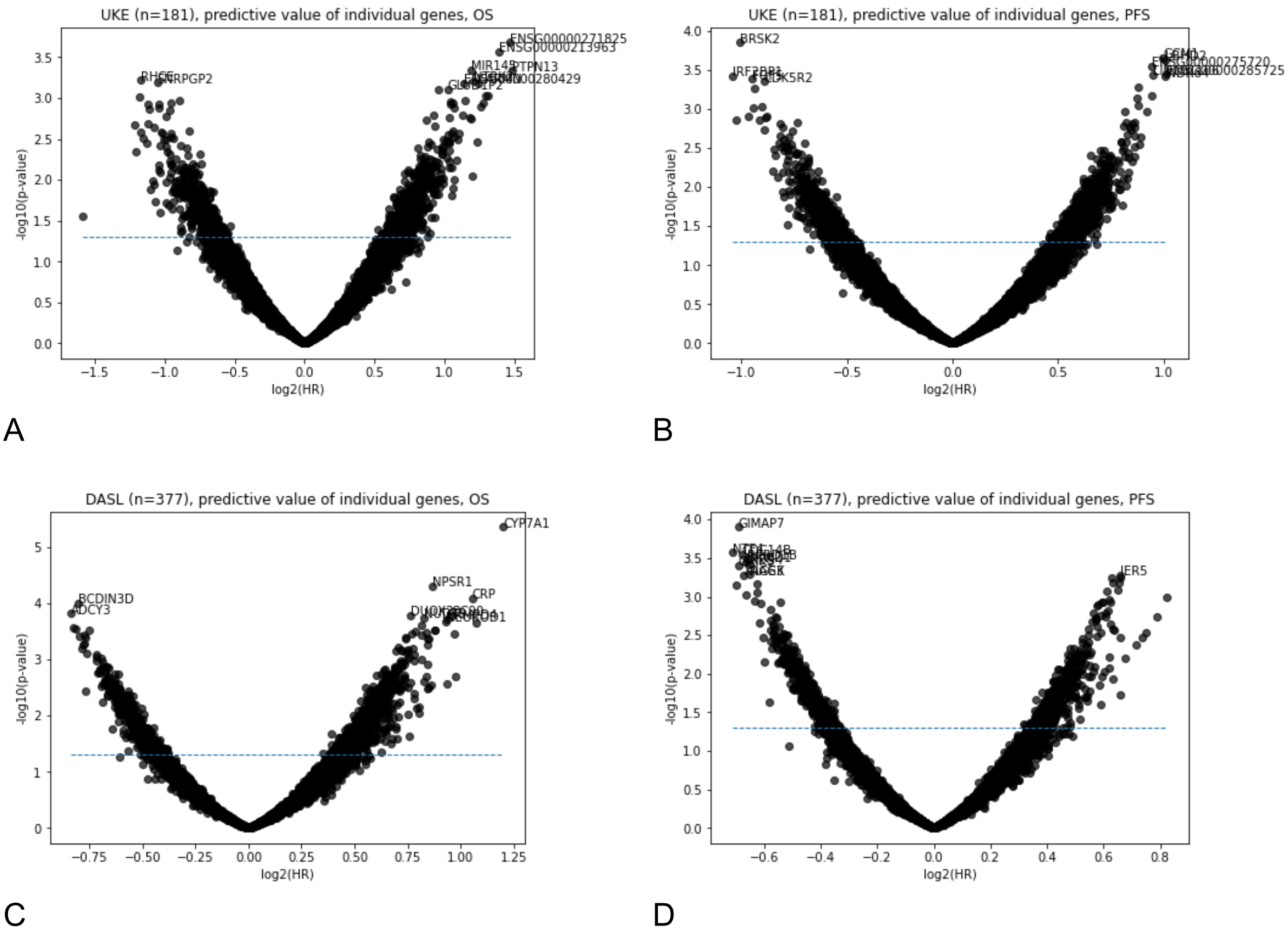

Fig. S2. Predictive values of individual gene expressions for overall survival (OS; **A,C**) and recurrence-free survival (RFS; **B,D**) in the UKE (**A,B**) and DASL (**C,D**) cohorts. Not adjusted p-values for interaction between treatment and gene expression are reported. After correction for multiple testing, all adjusted p-values exceeded 0.93 for both OS and PFS in the UKE, and 0.09 in the DASL.

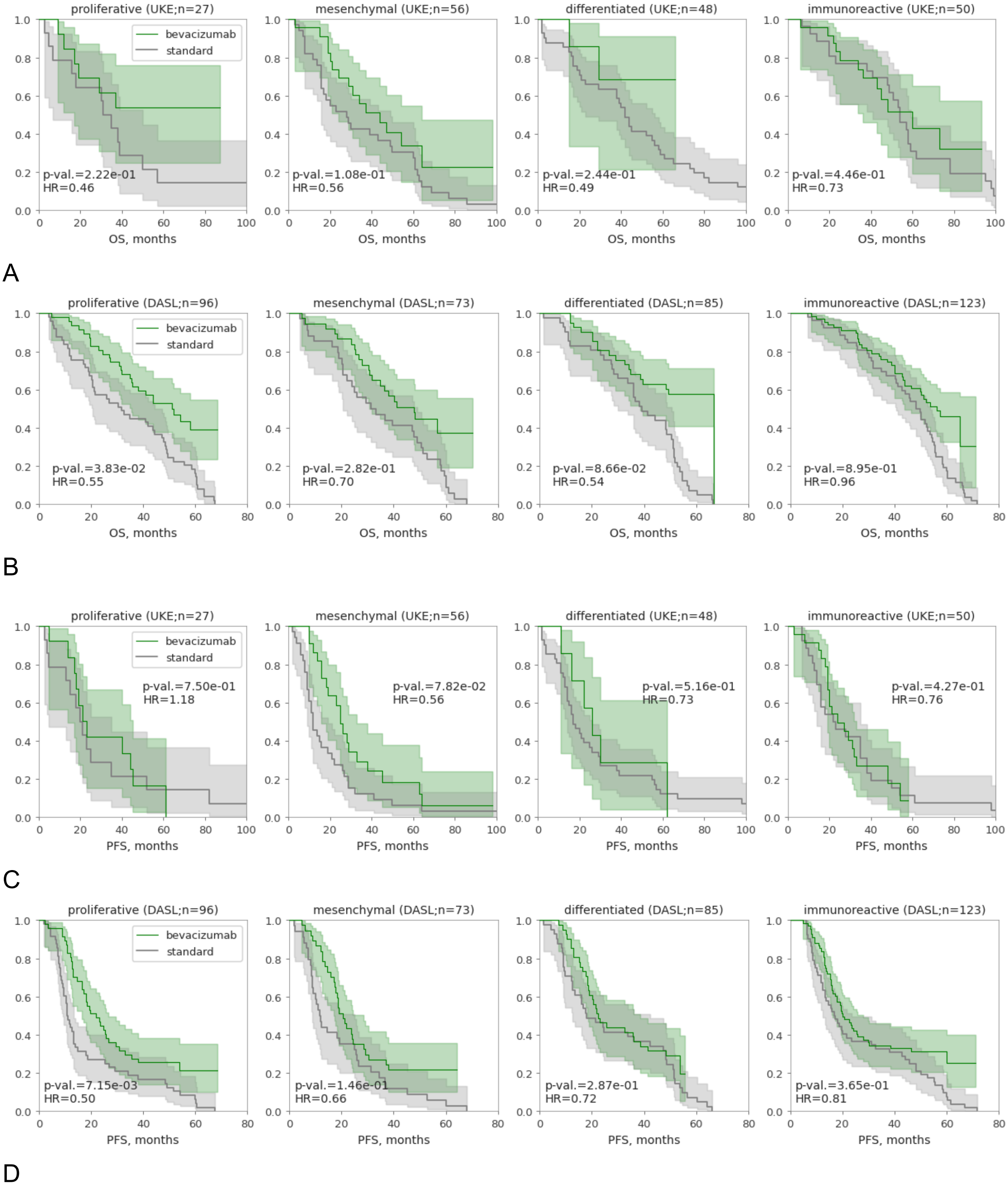

Fig. S3. Kaplan-Meier plots for OS (**A,B**) and PFS (**C,D**) in the bevacizumab and standard treatment groups in the UKE (**A,C**) and DASL (**B,D**) cohorts stratified by molecular subtype. In the UKE cohort, molecular subtypes were determined by the *consensusOV* classifier. In the DASL cohort, molecular subtypes reported by Kommos et al. were used. P-values not adjusted for multiple testing are reported.

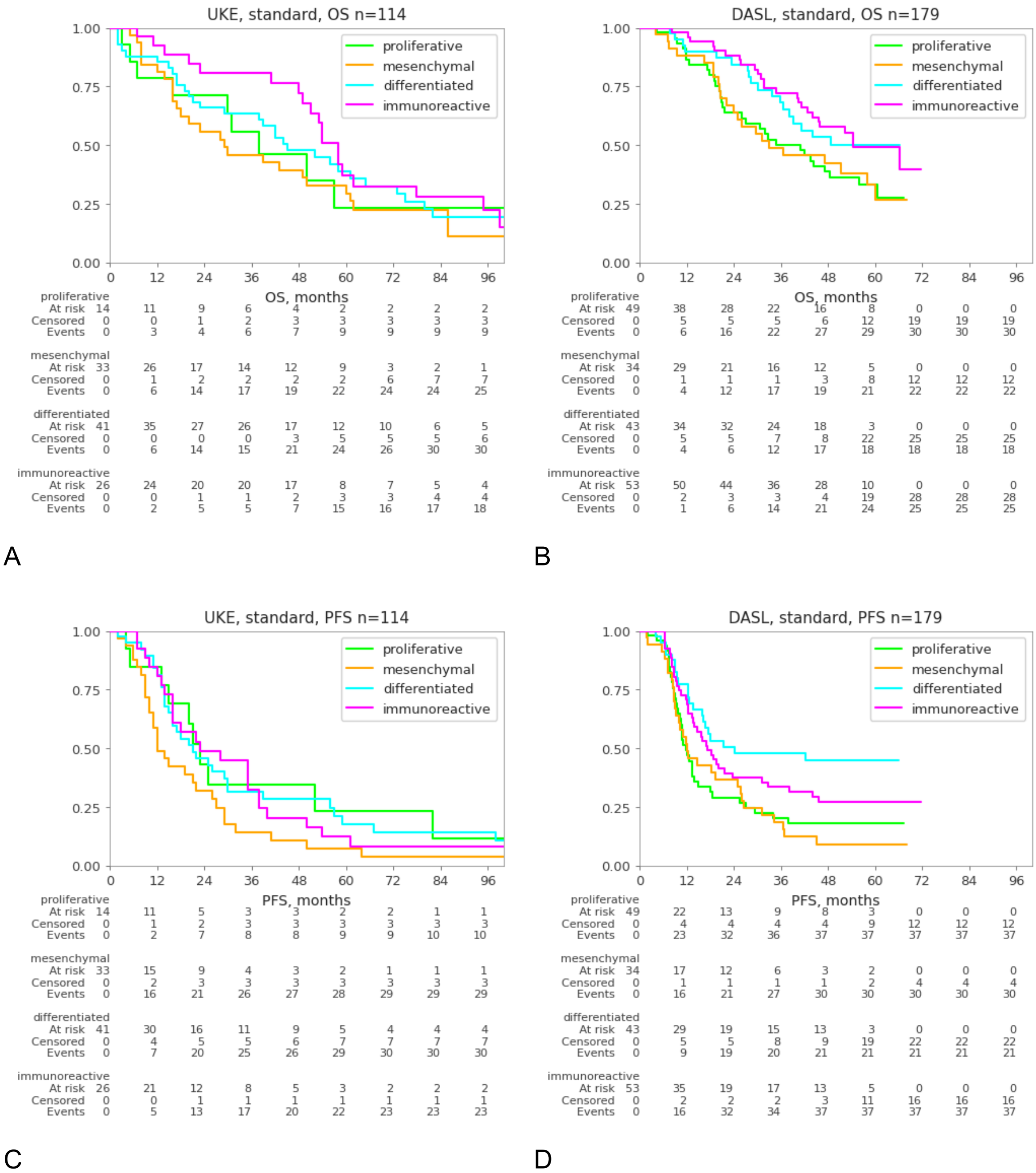

Fig. S4. Kaplan-Meier plots for OS (**A,B**) and PFS (**C,D**) for patients with tumors of different molecular subtypes under **standard treatment** in the UKE (**A,C**), DASL (**B,D**).

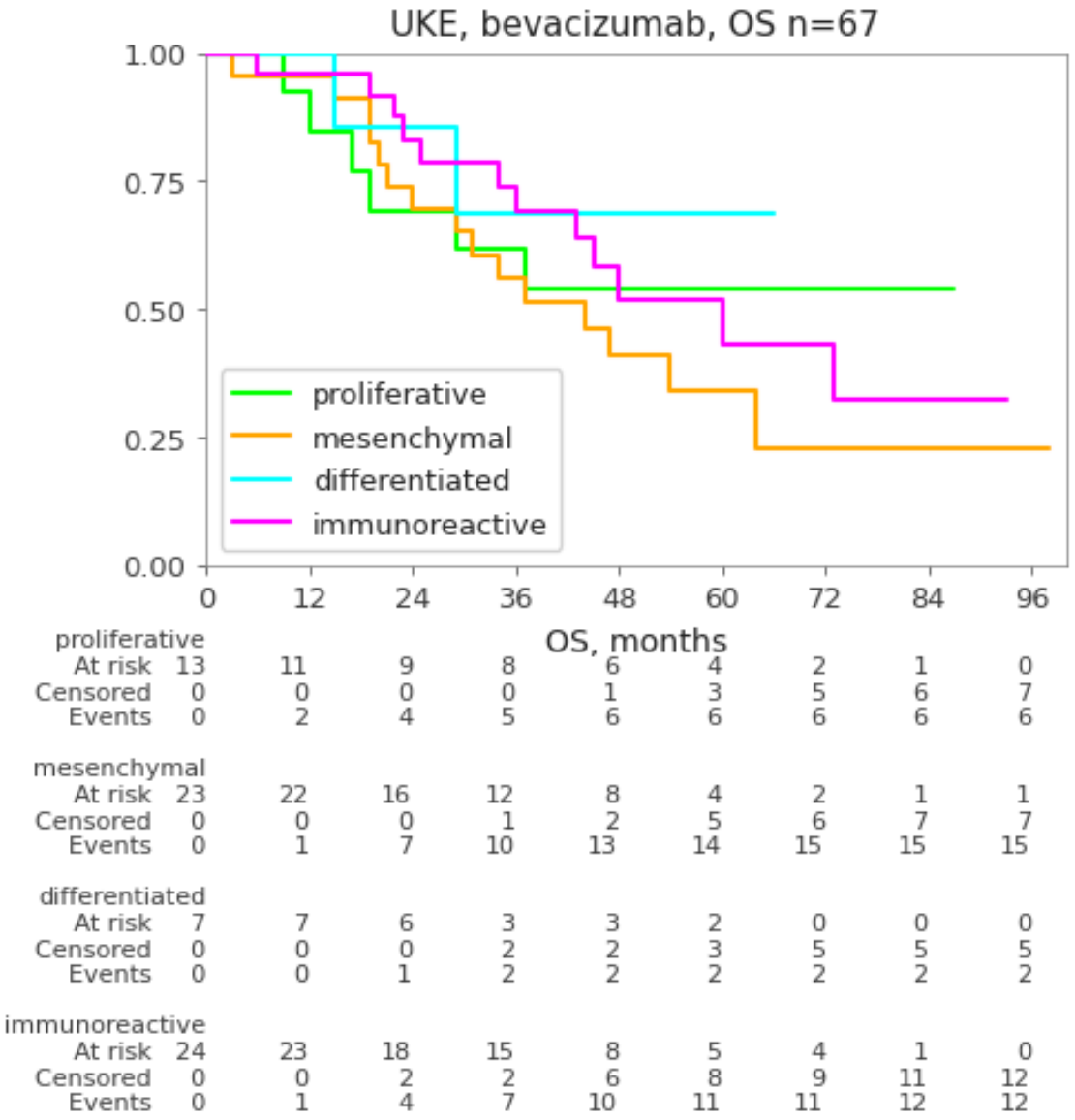

A

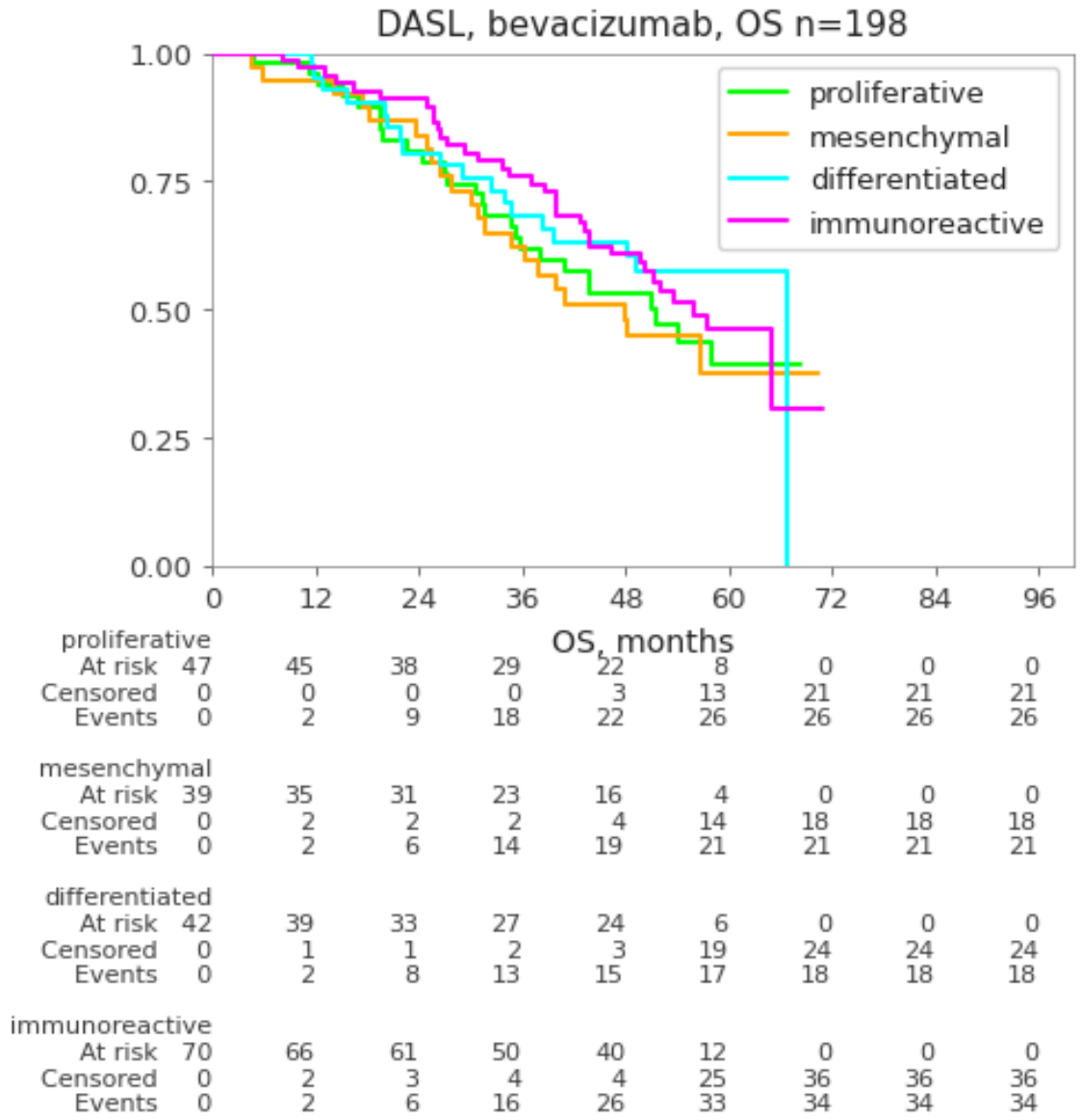

B

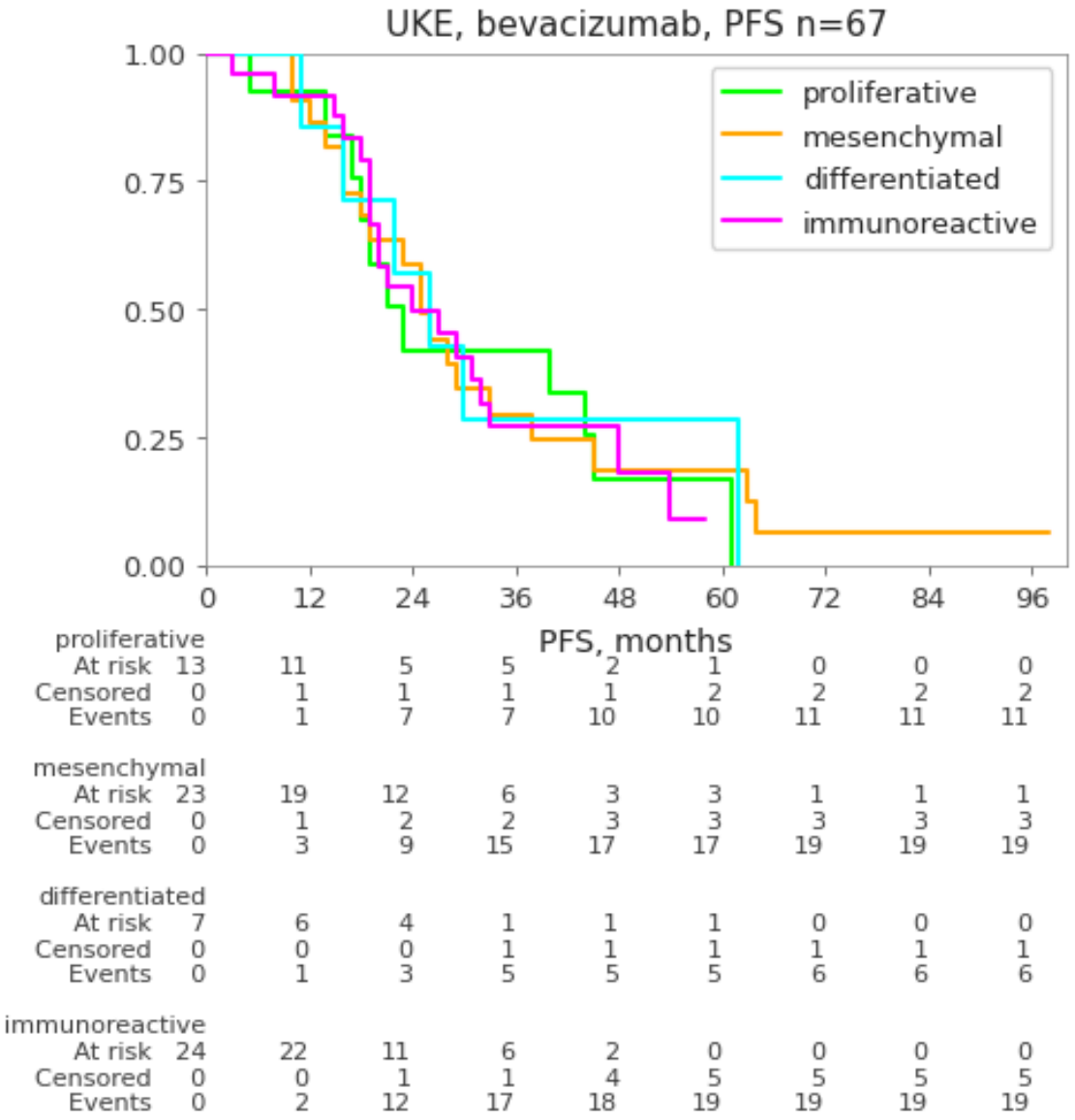

C

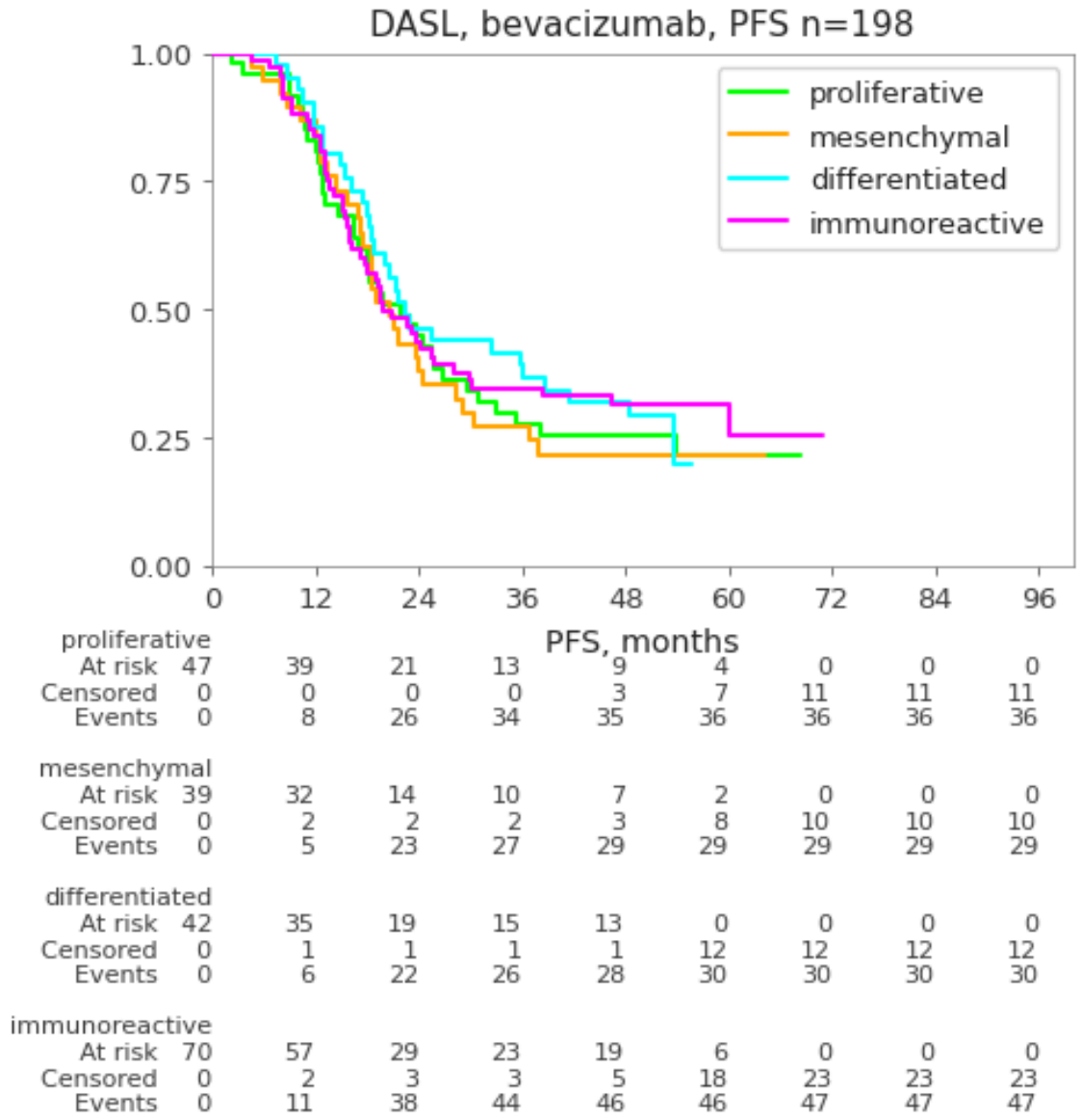

D

Fig. S5. Kaplan Meier plots for OS (**A,B**) and PFS (**C,D**) for patients with tumors of different molecular subtypes under under **bevacizumab treatment** in the UKE (**A,C**) and DASL (**B,D**) cohorts.

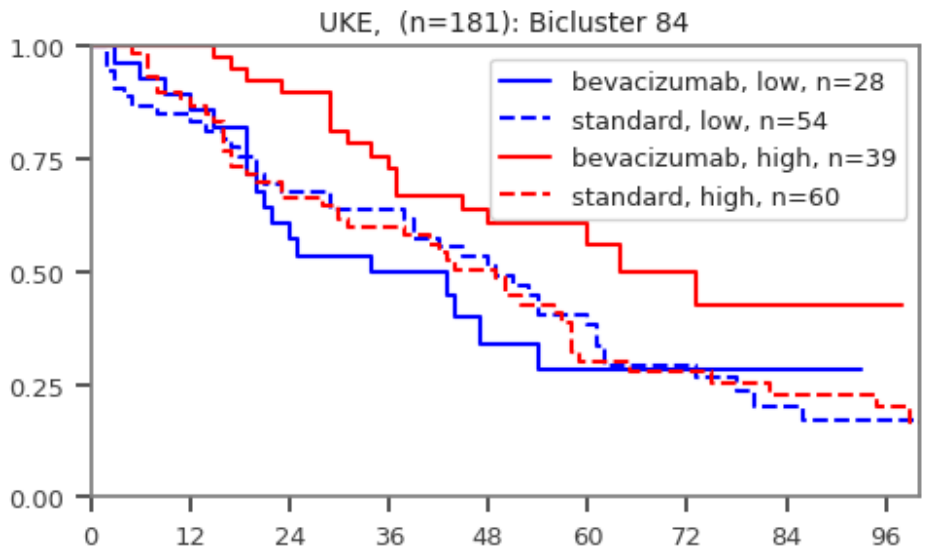

	0	12	24	36	48	60	72	84	96
**bevacizumab, low, n=28**									
At risk	28	24	16	13	6	4	1	1	0
Censored	0	0	0	1	5	6	9	9	10
Events	0	4	12	14	17	18	18	18	18
**standard, low, n=54**									
At risk	54	44	34	32	24	17	11	6	5
Censored	0	1	3	3	5	6	8	10	10
Events	0	9	17	19	25	31	35	38	39
**bevacizumab, high, n=39**									
At risk	39	39	33	25	19	11	7	2	1
Censored	0	0	2	4	6	13	16	20	21
Events	0	0	4	10	14	15	16	17	17
**standard, high, n=60**									
At risk	60	52	39	34	26	14	11	9	7
Censored	0	0	1	2	5	7	9	9	10
Events	0	8	20	24	29	39	40	42	43

A

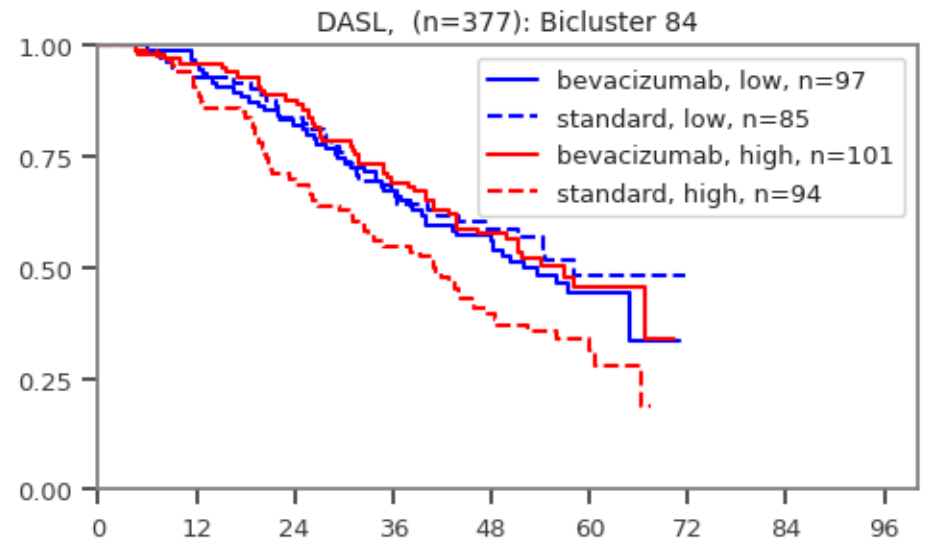

	0	12	24	36	48	60	72	84	96
**bevacizumab, low, n=97**									
At risk	97	91	78	62	49	13	0	0	0
Censored	0	2	2	4	7	35	47	47	47
Events	0	4	17	31	41	49	50	50	50
**standard, low, n=85**									
At risk	85	74	66	51	41	12	0	0	0
Censored	0	5	6	8	12	37	49	49	49
Events	0	6	13	26	32	36	36	36	36
**bevacizumab, high, n=101**									
At risk	101	94	85	67	53	17	0	0	0
Censored	0	3	4	4	7	36	52	52	52
Events	0	4	12	30	41	48	49	49	49
**standard, high, n=94**									
At risk	94	77	59	47	33	14	0	0	0
Censored	0	8	8	8	9	24	35	35	35
Events	0	9	27	39	52	56	59	59	59

B

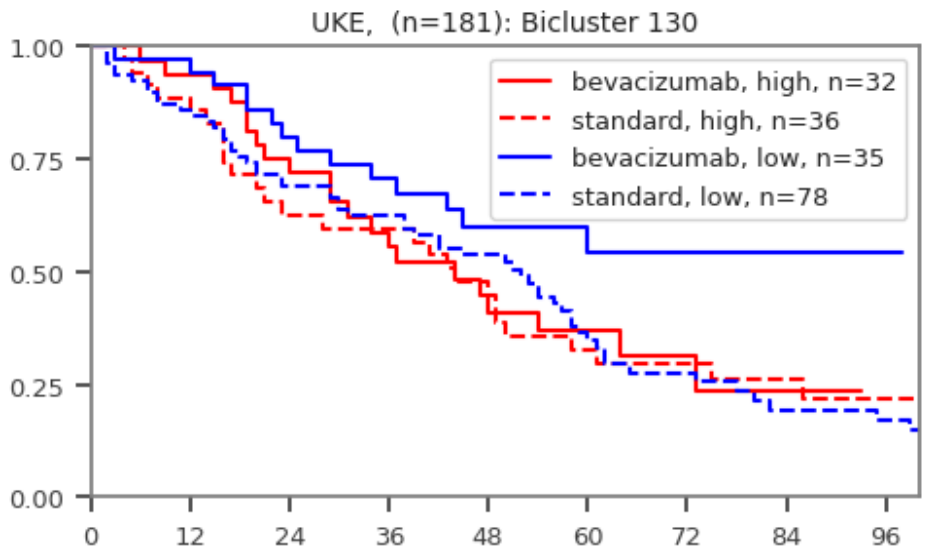

	0	12	24	36	48	60	72	84	96
**bevacizumab, high, n=32**									
At risk	32	30	22	16	11	7	4	1	0
Censored	0	0	1	2	3	6	8	10	11
Events	0	2	9	14	18	19	20	21	21
**standard, high, n=36**									
At risk	36	30	21	20	15	11	8	6	4
Censored	0	1	2	2	2	2	4	5	6
Events	0	5	13	14	19	23	24	25	26
**bevacizumab, low, n=35**									
At risk	35	33	27	22	14	8	4	2	1
Censored	0	0	1	3	8	13	17	19	20
Events	0	2	7	10	13	14	14	14	14
**standard, low, n=78**									
At risk	78	66	52	46	35	20	14	9	8
Censored	0	0	2	3	8	11	13	14	14
Events	0	12	24	29	35	47	51	55	56

C

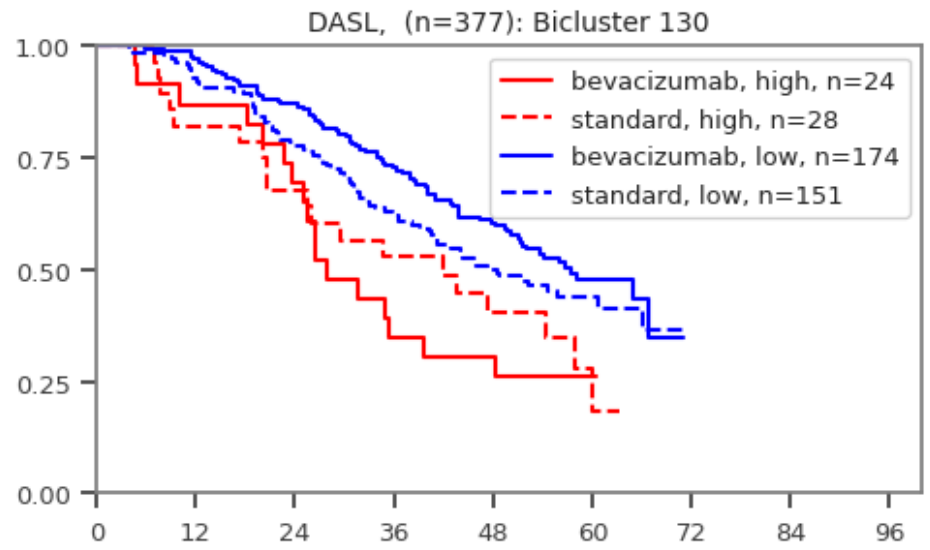

	0	12	24	36	48	60	72	84	96
**bevacizumab, high, n=24**									
At risk	24	20	16	8	7	1	0	0	0
Censored	0	1	1	1	1	6	7	7	7
Events	0	3	7	15	16	17	17	17	17
**standard, high, n=28**									
At risk	28	23	18	14	10	3	0	0	0
Censored	0	0	1	1	2	7	9	9	9
Events	0	5	9	13	16	18	19	19	19
**bevacizumab, low, n=174**									
At risk	174	165	147	121	95	29	0	0	0
Censored	0	4	5	7	13	65	92	92	92
Events	0	5	22	46	66	80	82	82	82
**standard, low, n=151**									
At risk	151	128	107	84	64	23	0	0	0
Censored	0	13	13	15	19	54	75	75	75
Events	0	10	31	52	68	74	76	76	76

D

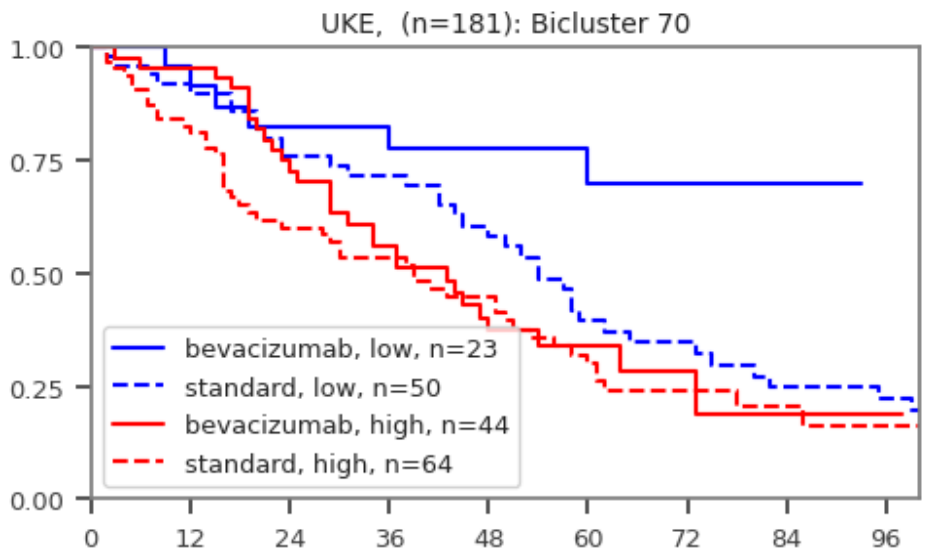

	0	12	24	36	48	60	72	84	96
**bevacizumab, low, n=23**									
At risk	23	21	18	15	12	8	5	2	0
Censored	0	0	1	3	6	9	12	15	17
Events	0	2	4	5	5	6	6	6	6
**standard, low, n=50**									
At risk	50	45	37	34	25	16	14	10	8
Censored	0	0	1	2	5	6	6	6	7
Events	0	5	12	14	20	28	30	34	35
**bevacizumab, high, n=44**									
At risk	44	42	31	23	13	7	3	1	1
Censored	0	0	1	2	5	10	13	14	14
Events	0	2	12	19	26	27	28	29	29
**standard, high, n=64**									
At risk	64	51	36	32	25	15	8	5	4
Censored	0	1	3	3	5	7	11	13	13
Events	0	12	25	29	34	42	45	46	47

E

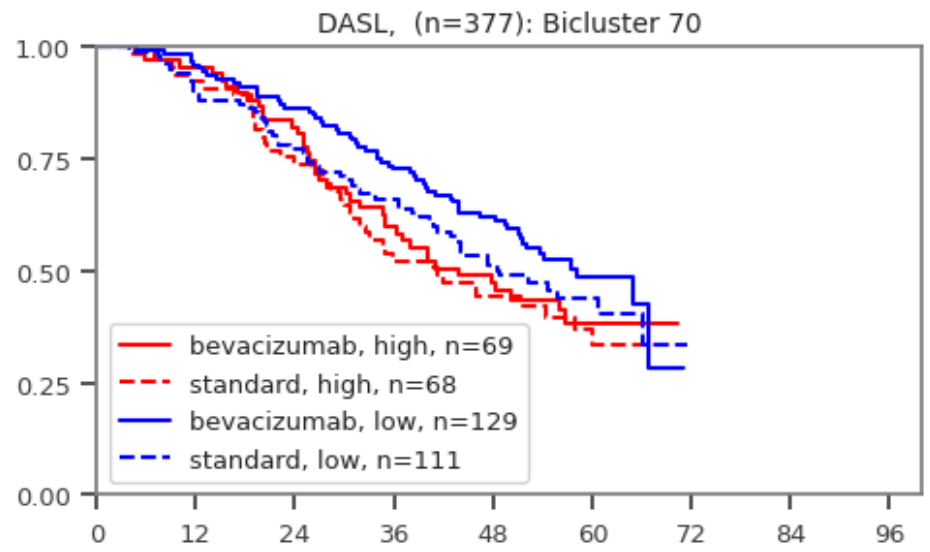

	0	12	24	36	48	60	72	84	96
**bevacizumab, high, n=69**									
At risk	69	64	55	40	29	11	0	0	0
Censored	0	2	2	2	5	19	30	30	30
Events	0	3	12	27	35	39	39	39	39
**standard, high, n=68**									
At risk	68	60	48	34	27	11	0	0	0
Censored	0	3	3	3	5	18	28	28	28
Events	0	5	17	31	36	39	40	40	40
**bevacizumab, low, n=129**									
At risk	129	121	108	89	73	19	0	0	0
Censored	0	3	4	6	9	52	69	69	69
Events	0	5	17	34	47	58	60	60	60
**standard, low, n=111**									
At risk	111	91	77	64	47	15	0	0	0
Censored	0	10	11	13	16	43	56	56	56
Events	0	10	23	34	48	53	55	55	55

F

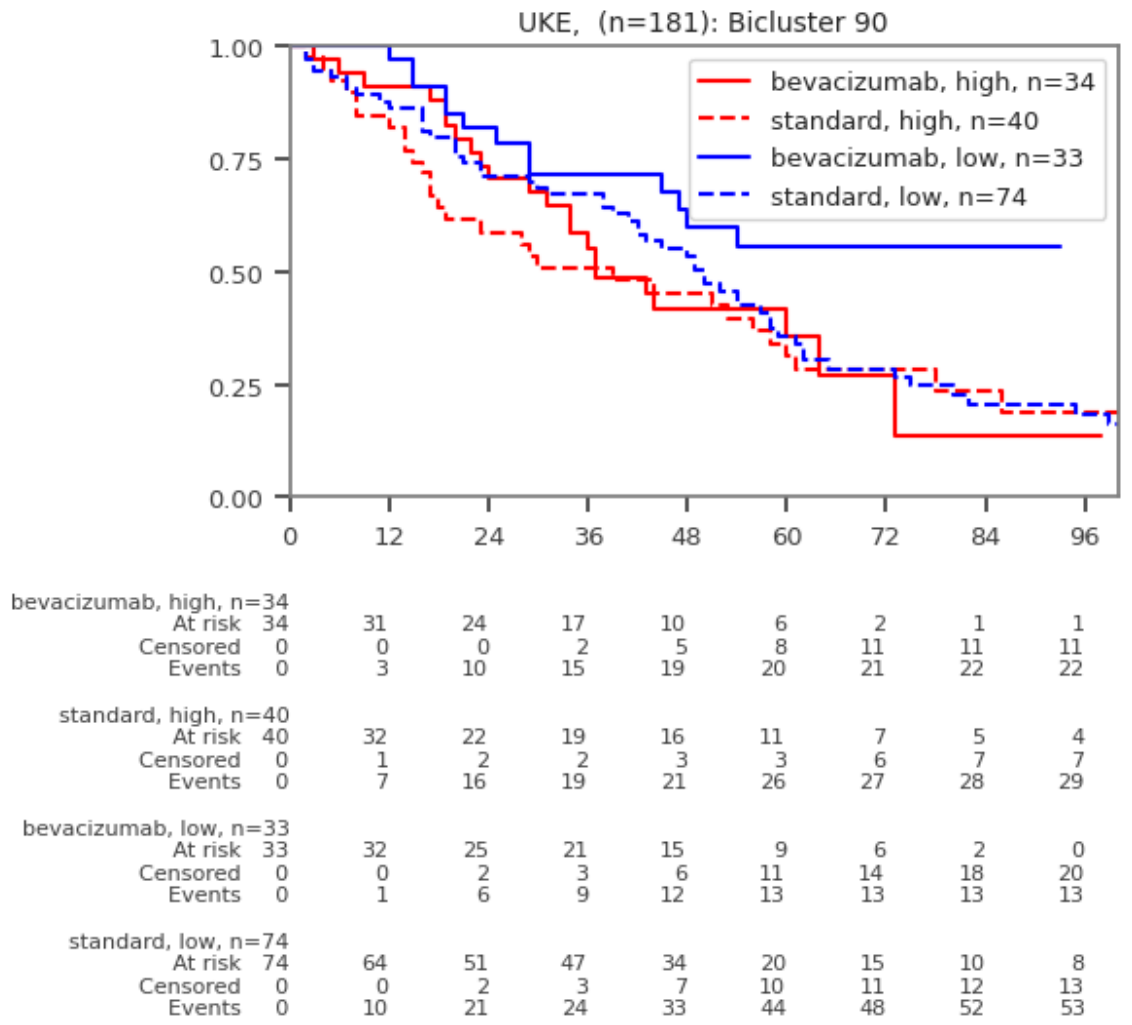

G

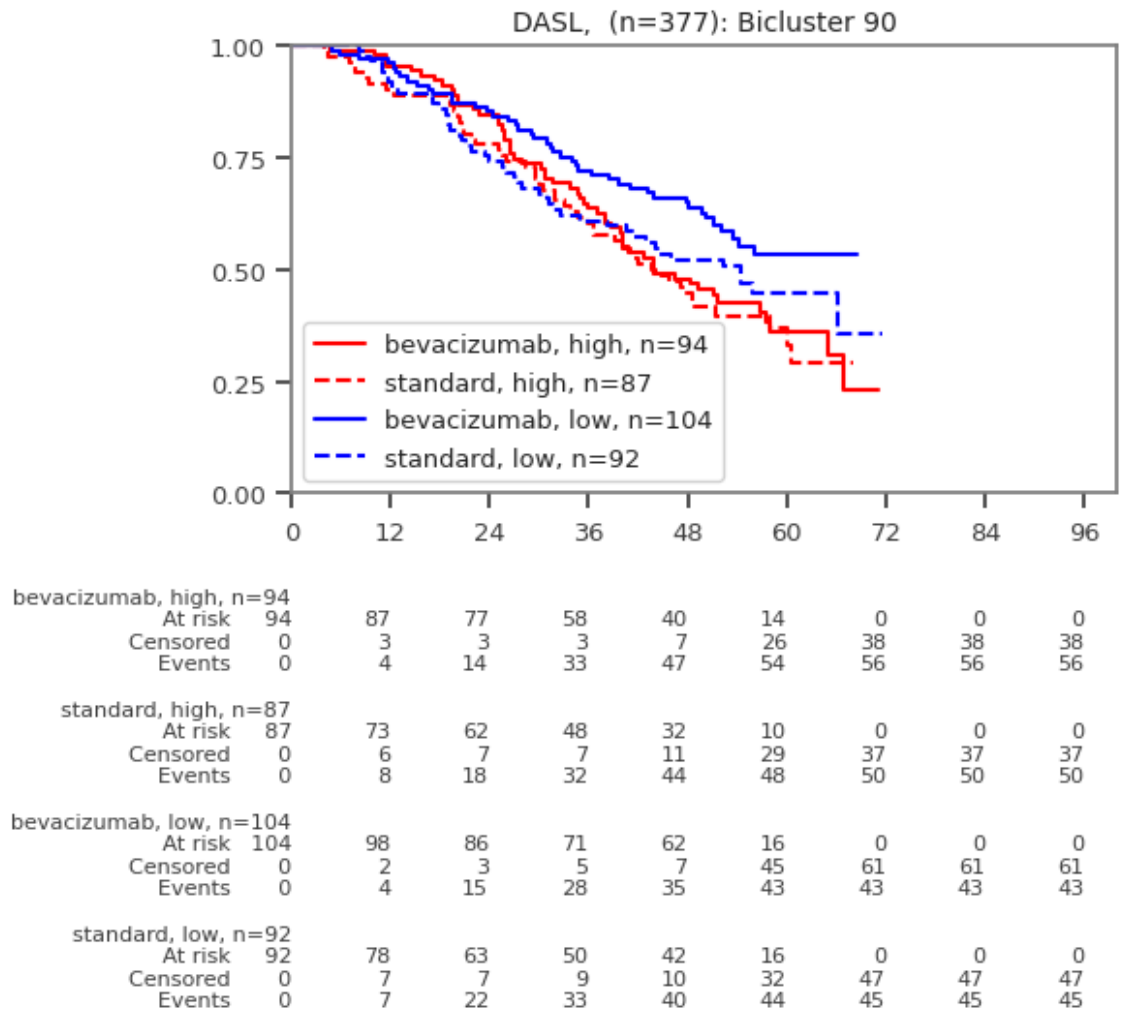

H

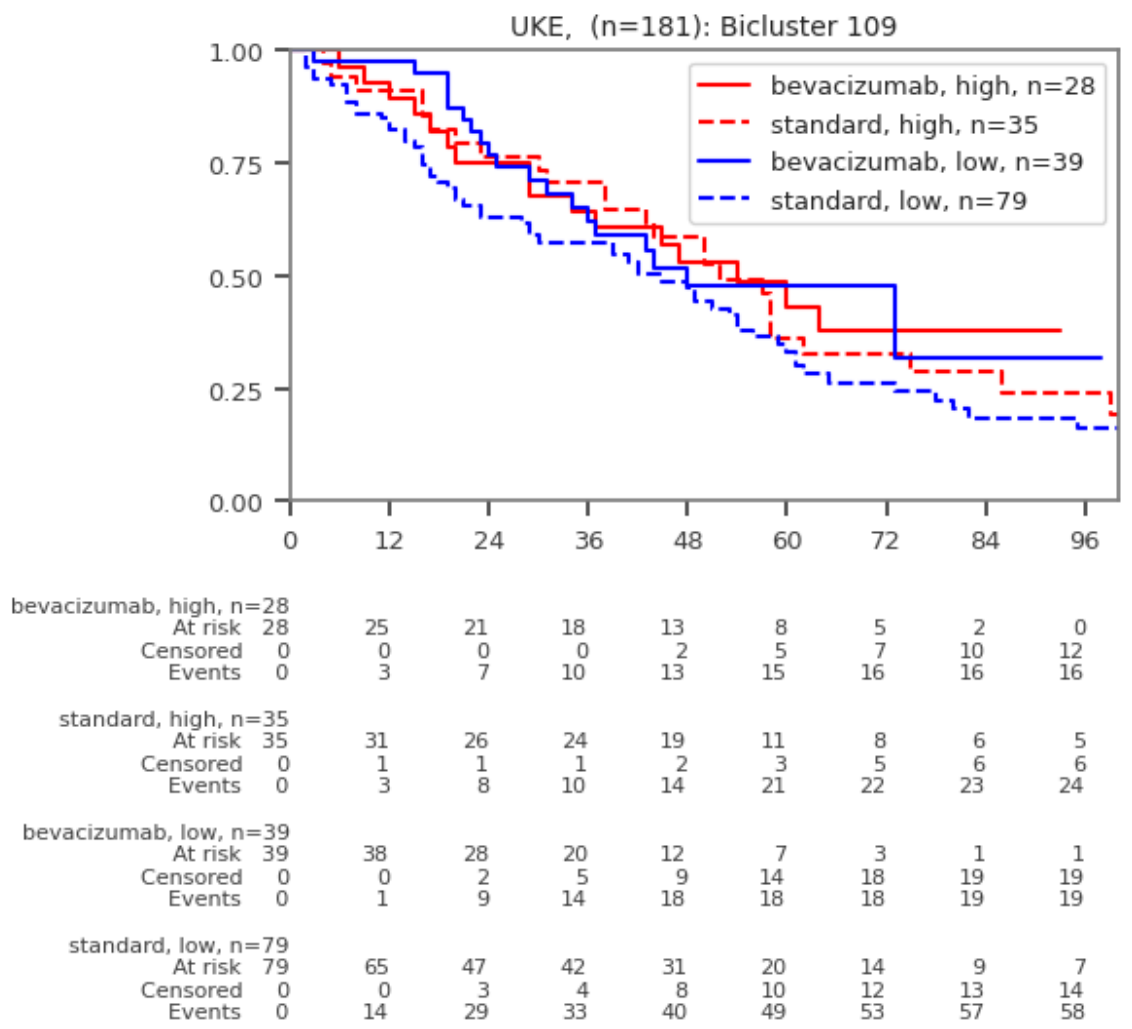

K

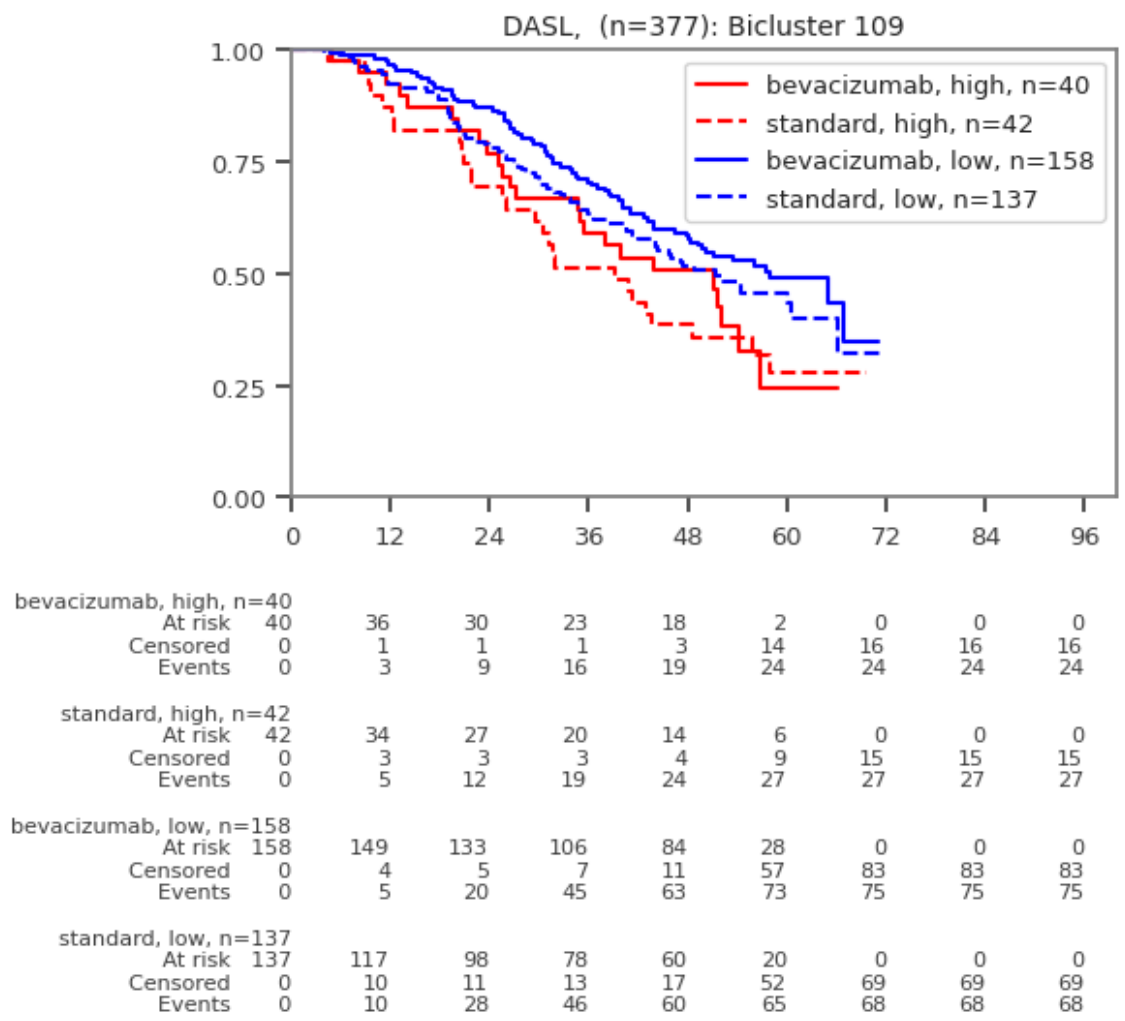

L

Fig. S6. Kaplan-Meier plots for **OS** of patient subgroups of the UKE and DASL cohorts stratified by treatment (solid line - bevacizumab; dashed line - standard chemotherapy) and expression of genes comprising biclusters 84 (**A,B**), 130 (**C,D**), 70 (**E,F**), 90 (**G,H**), and 109 (**K,L**) prioritized by random survival forests as the best predictors of **OS**.

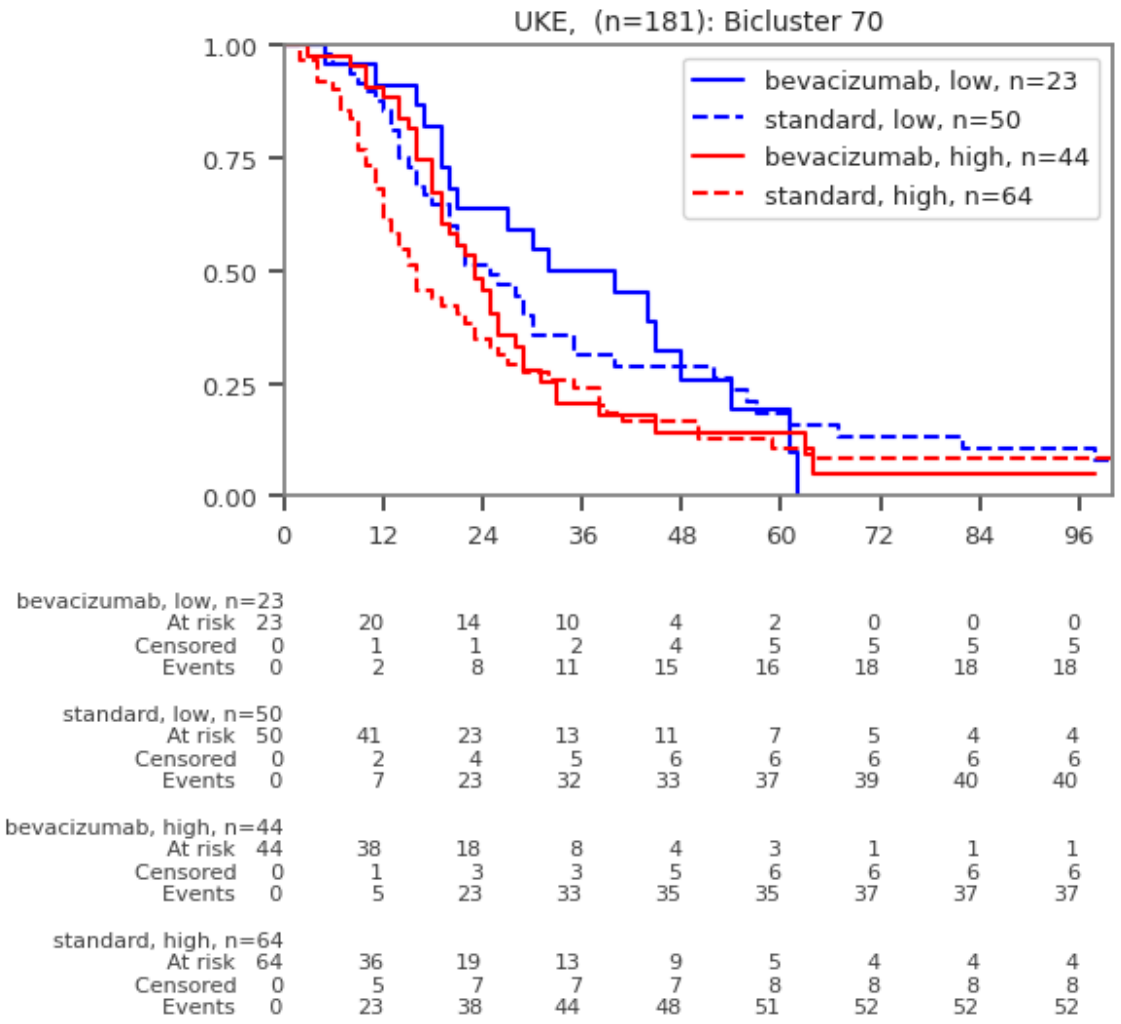
UKE, (n=181): Bicluster 70
1.00
0.75
0.50
0.25
0.00
0 12 24 36 48 60 72 84 96
bevacizumab, low, n=23
standard, low, n=50
bevacizumab, high, n=44
standard, high, n=64
bevacizumab, low, n=23
At risk 23 20 14 10 4 2 0 0 0
Censored 0 1 1 2 4 5 5 5 5
Events 0 2 8 11 15 16 18 18 18
standard, low, n=50
At risk 50 41 23 13 11 7 5 4 4
Censored 0 2 4 5 6 6 6 6 6
Events 0 7 23 32 33 37 39 40 40
bevacizumab, high, n=44
At risk 44 38 18 8 4 3 1 1 1
Censored 0 1 3 3 5 6 6 6 6
Events 0 5 23 33 35 35 37 37 37
standard, high, n=64
At risk 64 36 19 13 9 5 4 4 4
Censored 0 5 7 7 7 8 8 8 8
Events 0 23 38 44 48 51 52 52 52

A

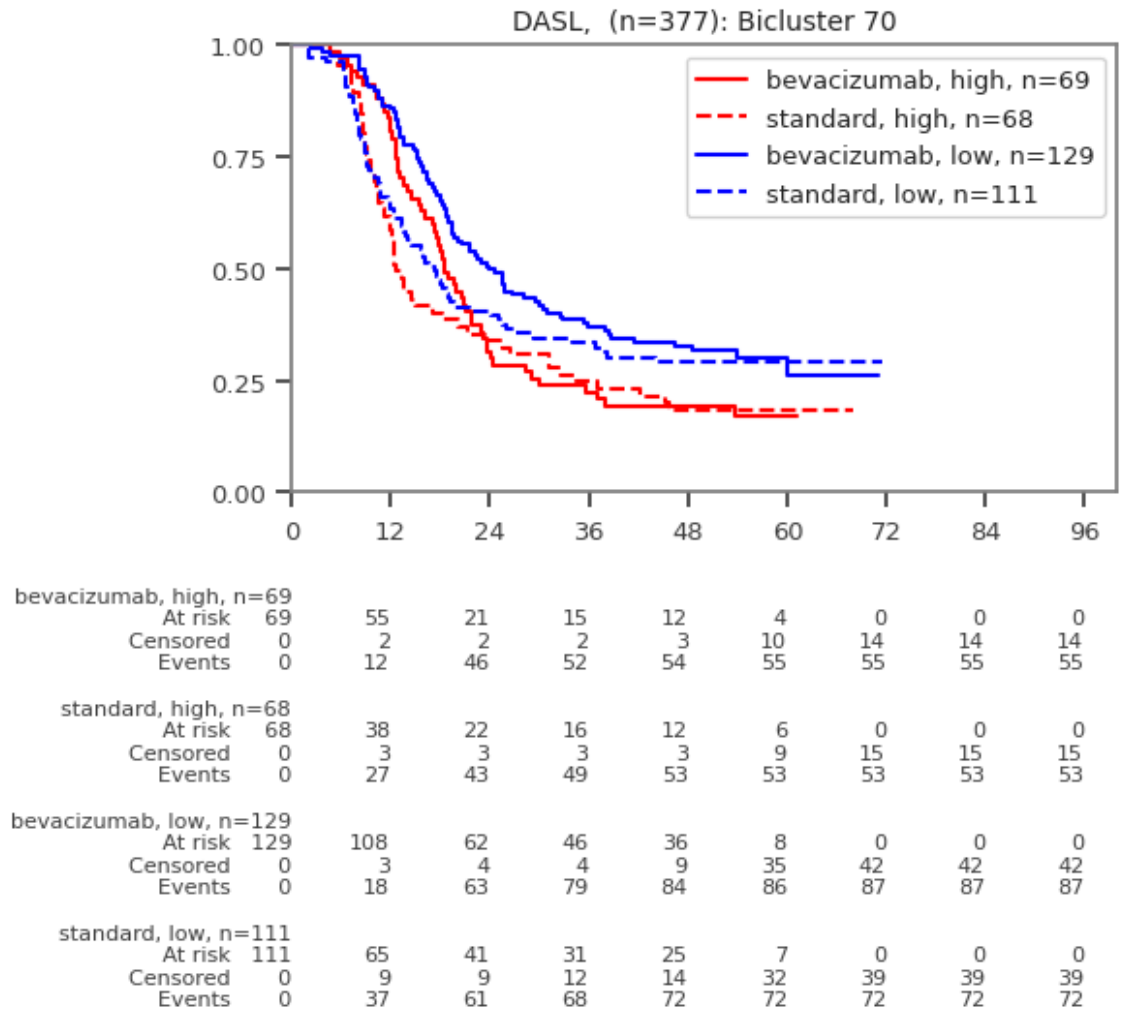
DASL, (n=377): Bicluster 70
1.00
0.75
0.50
0.25
0.00
0 12 24 36 48 60 72 84 96
bevacizumab, high, n=69
standard, high, n=68
bevacizumab, low, n=129
standard, low, n=111
bevacizumab, high, n=69
At risk 69 55 21 15 12 4 0 0 0
Censored 0 2 2 2 3 10 14 14 14
Events 0 12 46 52 54 55 55 55 55
standard, high, n=68
At risk 68 38 22 16 12 6 0 0 0
Censored 0 3 3 3 3 9 15 15 15
Events 0 27 43 49 53 53 53 53 53
bevacizumab, low, n=129
At risk 129 108 62 46 36 8 0 0 0
Censored 0 3 4 4 9 35 42 42 42
Events 0 18 63 79 84 86 87 87 87
standard, low, n=111
At risk 111 65 41 31 25 7 0 0 0
Censored 0 9 9 12 14 32 39 39 39
Events 0 37 61 68 72 72 72 72 72

B

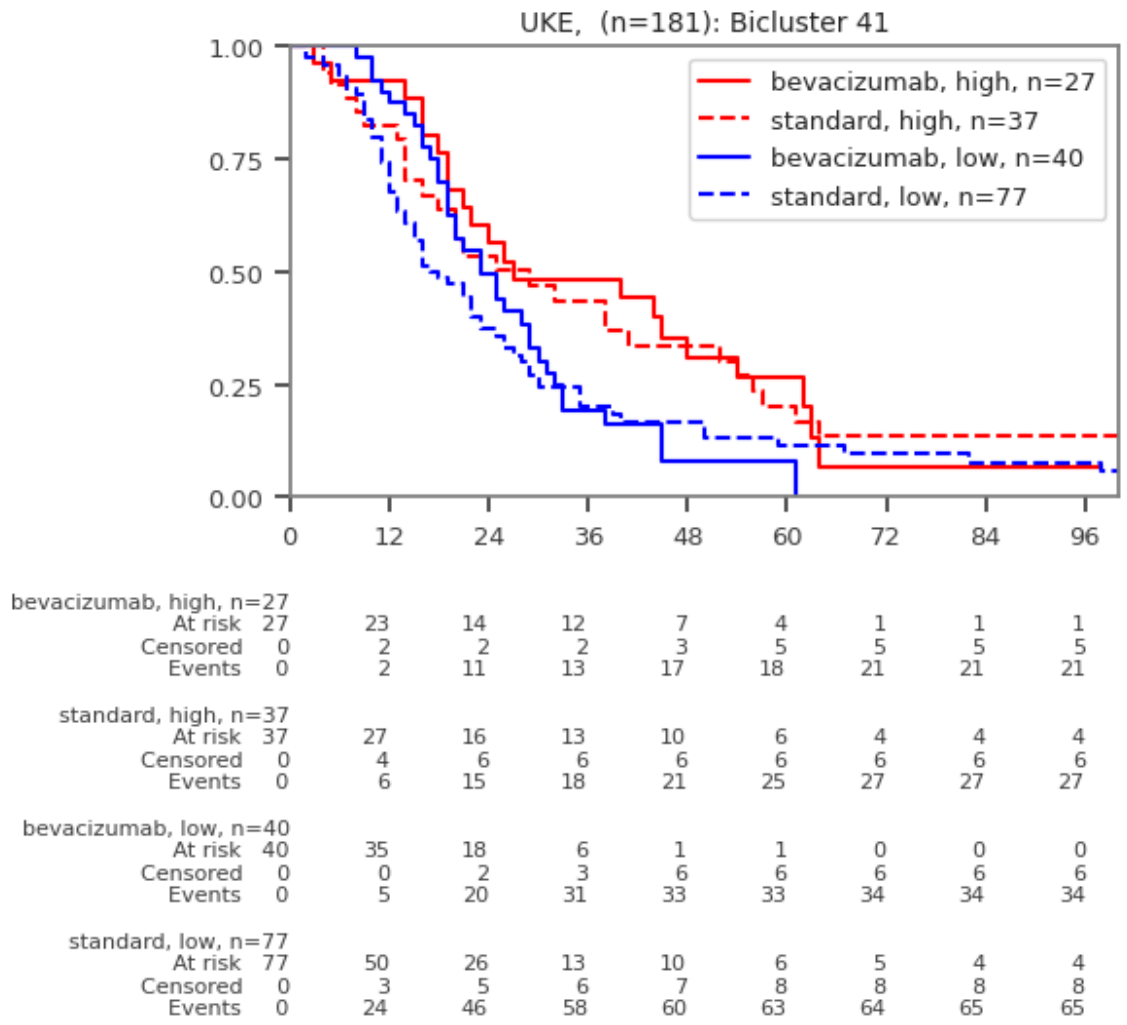
UKE, (n=181): Bicluster 41
1.00
0.75
0.50
0.25
0.00
0 12 24 36 48 60 72 84 96
bevacizumab, high, n=27
standard, high, n=37
bevacizumab, low, n=40
standard, low, n=77
bevacizumab, high, n=27
At risk 27 23 14 12 7 4 1 1 1
Censored 0 2 2 2 3 5 5 5 5
Events 0 2 11 13 17 18 21 21 21
standard, high, n=37
At risk 37 27 16 13 10 6 4 4 4
Censored 0 4 6 6 6 6 6 6 6
Events 0 6 15 18 21 25 27 27 27
bevacizumab, low, n=40
At risk 40 35 18 6 1 1 0 0 0
Censored 0 0 2 3 6 6 6 6 6
Events 0 5 20 31 33 33 34 34 34
standard, low, n=77
At risk 77 50 26 13 10 6 5 4 4
Censored 0 3 5 6 7 8 8 8 8
Events 0 24 46 58 60 63 64 65 65

C

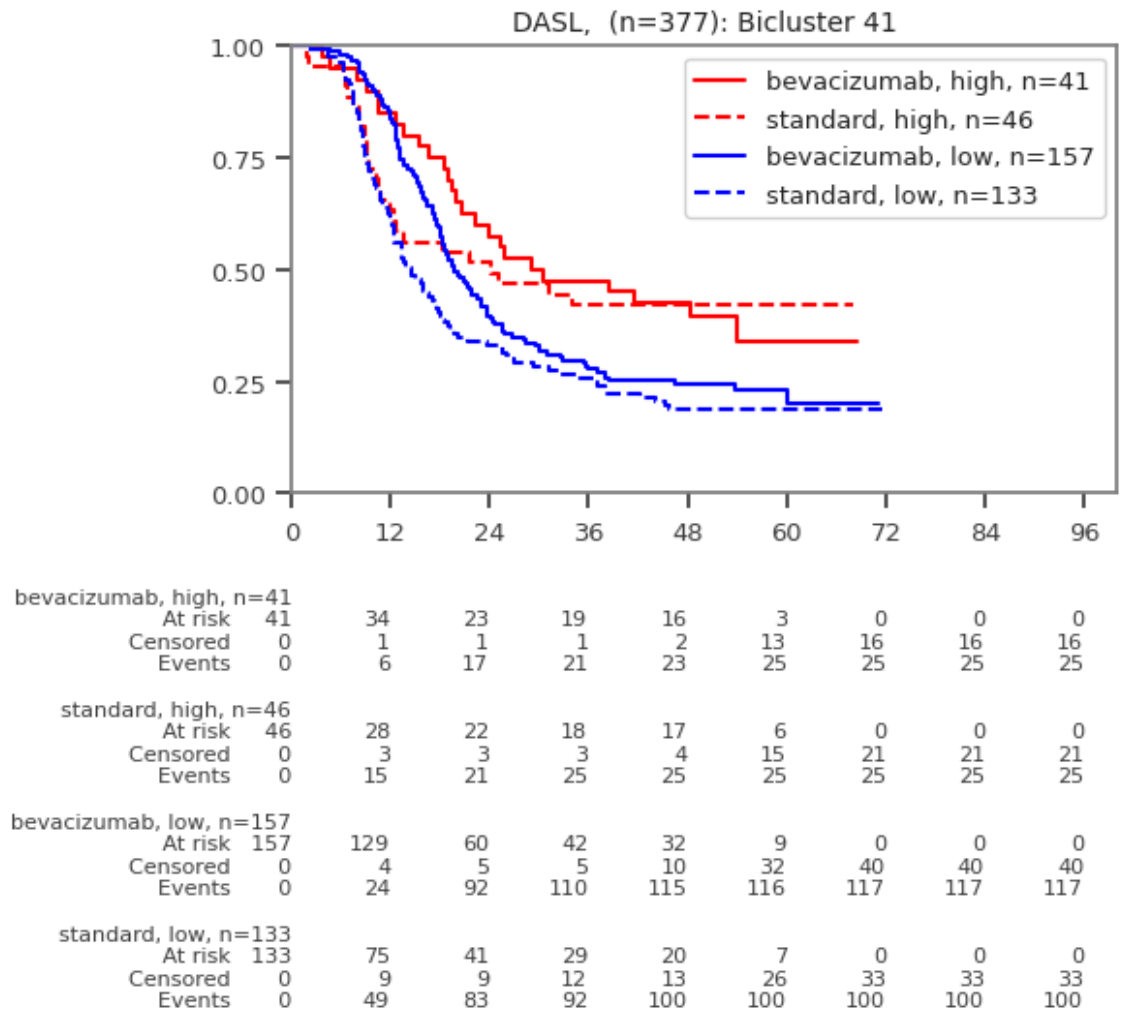
DASL, (n=377): Bicluster 41
1.00
0.75
0.50
0.25
0.00
0 12 24 36 48 60 72 84 96
bevacizumab, high, n=41
standard, high, n=46
bevacizumab, low, n=157
standard, low, n=133
bevacizumab, high, n=41
At risk 41 34 23 19 16 3 0 0 0
Censored 0 1 1 1 2 13 16 16 16
Events 0 6 17 21 23 25 25 25 25
standard, high, n=46
At risk 46 28 22 18 17 6 0 0 0
Censored 0 3 3 3 4 15 21 21 21
Events 0 15 21 25 25 25 25 25 25
bevacizumab, low, n=157
At risk 157 129 60 42 32 9 0 0 0
Censored 0 4 5 5 10 32 40 40 40
Events 0 24 92 110 115 116 117 117 117
standard, low, n=133
At risk 133 75 41 29 20 7 0 0 0
Censored 0 9 9 12 13 26 33 33 33
Events 0 49 83 92 100 100 100 100 100

D

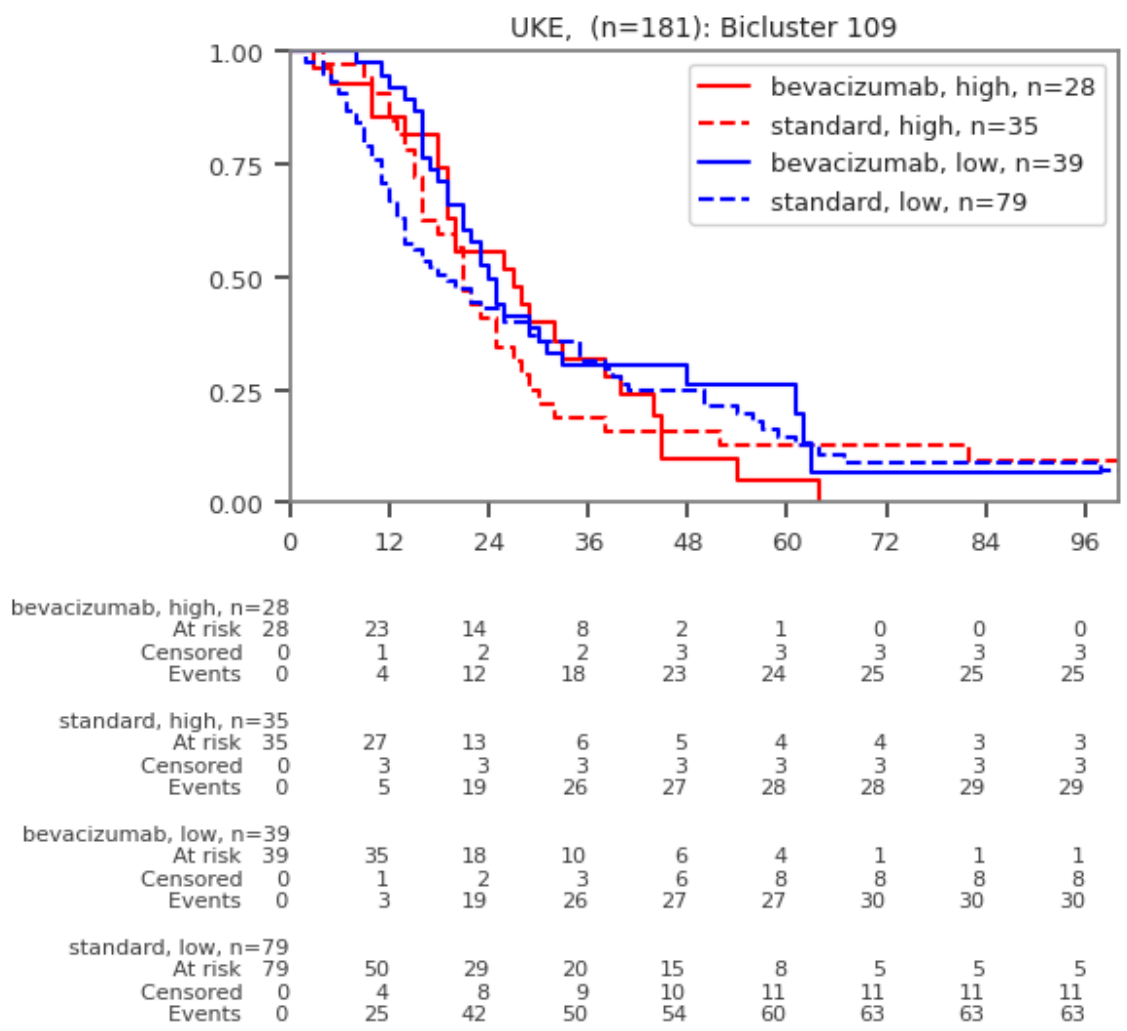
UKE, (n=181): Bicluster 109
1.00
0.75
0.50
0.25
0.00
0 12 24 36 48 60 72 84 96
bevacizumab, high, n=28
standard, high, n=35
bevacizumab, low, n=39
standard, low, n=79
bevacizumab, high, n=28
At risk 28 23 14 8 2 1 0 0 0
Censored 0 1 2 2 3 3 3 3 3
Events 0 4 12 18 23 24 25 25 25
standard, high, n=35
At risk 35 27 13 6 5 4 4 3 3
Censored 0 3 3 3 3 3 3 3 3
Events 0 5 19 26 27 28 28 29 29
bevacizumab, low, n=39
At risk 39 35 18 10 6 4 1 1 1
Censored 0 1 2 3 6 8 8 8 8
Events 0 3 19 26 27 27 30 30 30
standard, low, n=79
At risk 79 50 29 20 15 8 5 5 5
Censored 0 4 8 9 10 11 11 11 11
Events 0 25 42 50 54 60 63 63 63

E

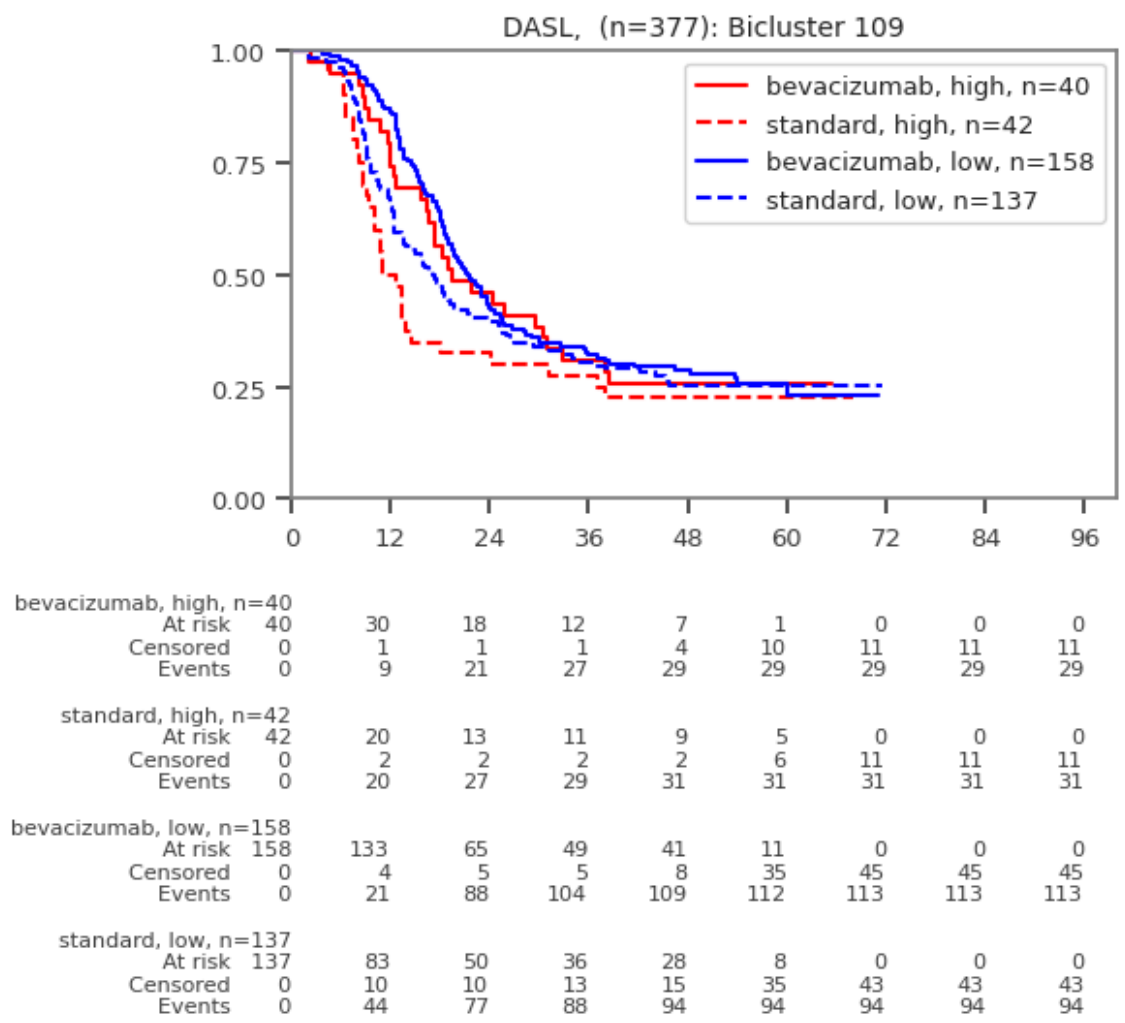
DASL, (n=377): Bicluster 109
1.00
0.75
0.50
0.25
0.00
0 12 24 36 48 60 72 84 96
bevacizumab, high, n=40
standard, high, n=42
bevacizumab, low, n=158
standard, low, n=137
bevacizumab, high, n=40
At risk 40 30 18 12 7 1 0 0 0
Censored 0 1 1 1 4 10 11 11 11
Events 0 9 21 27 29 29 29 29 29
standard, high, n=42
At risk 42 20 13 11 9 5 0 0 0
Censored 0 2 2 2 2 6 11 11 11
Events 0 20 27 29 31 31 31 31 31
bevacizumab, low, n=158
At risk 158 133 65 49 41 11 0 0 0
Censored 0 4 5 5 8 35 45 45 45
Events 0 21 88 104 109 112 113 113 113
standard, low, n=137
At risk 137 83 50 36 28 8 0 0 0
Censored 0 10 10 13 15 35 43 43 43
Events 0 44 77 88 94 94 94 94 94

F

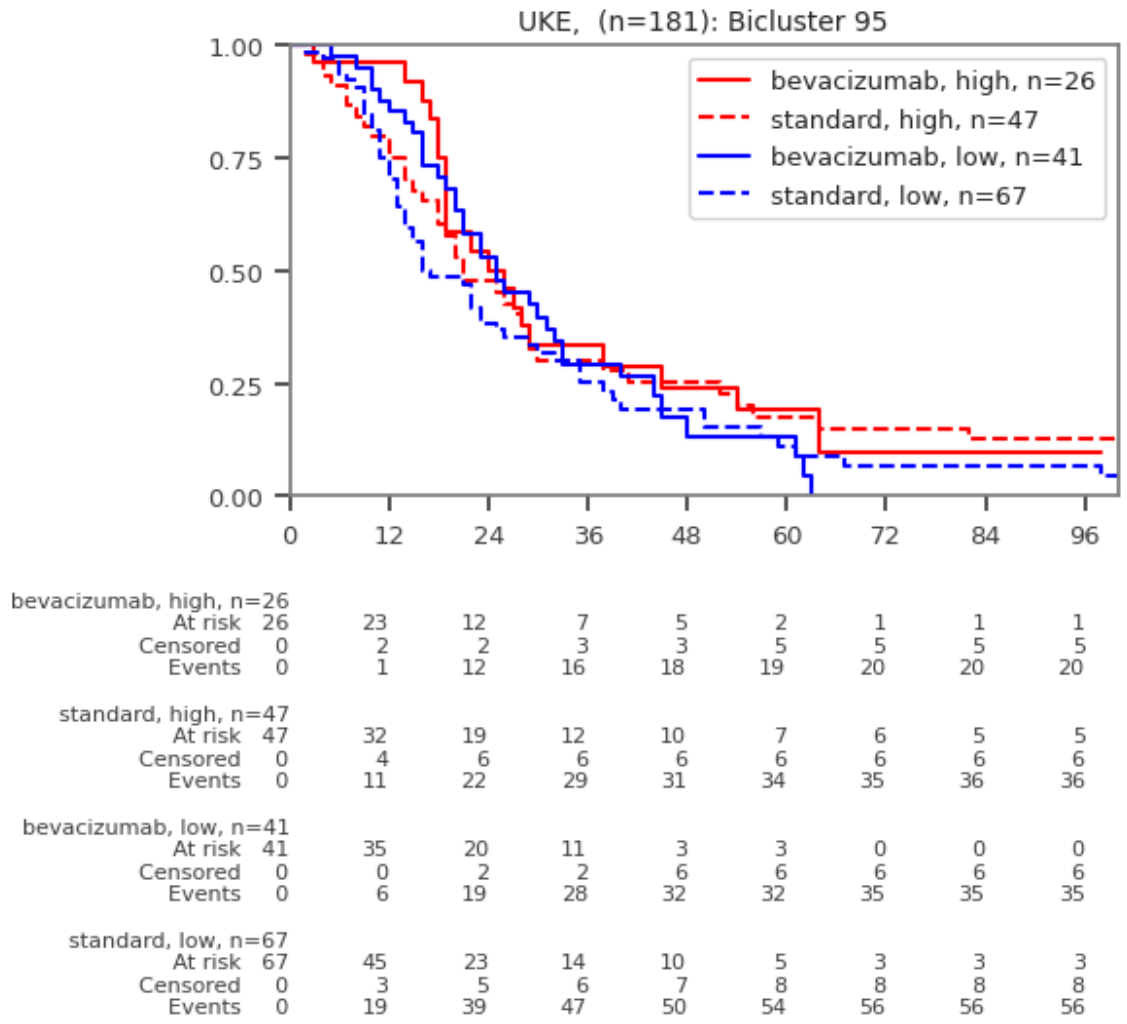

G

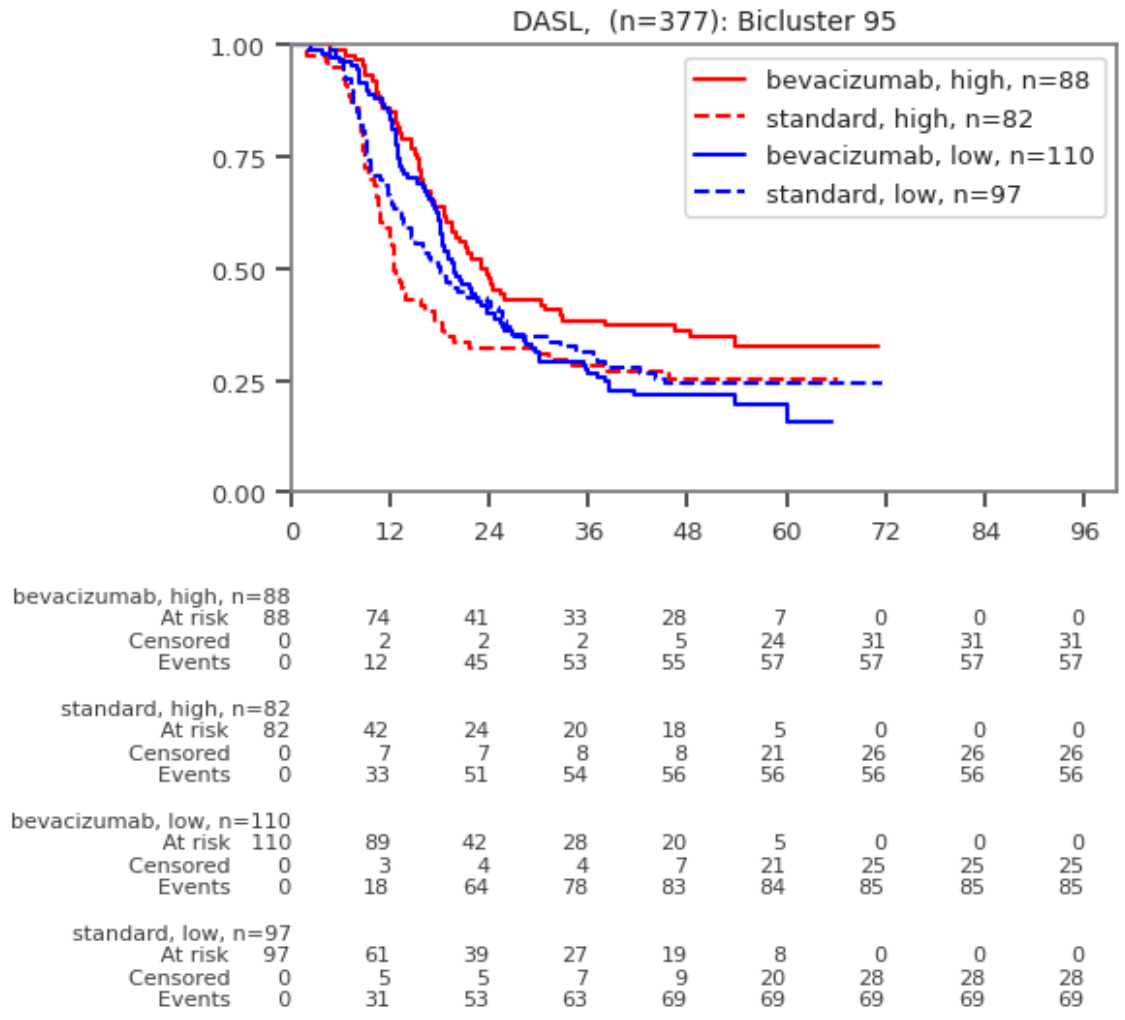

H

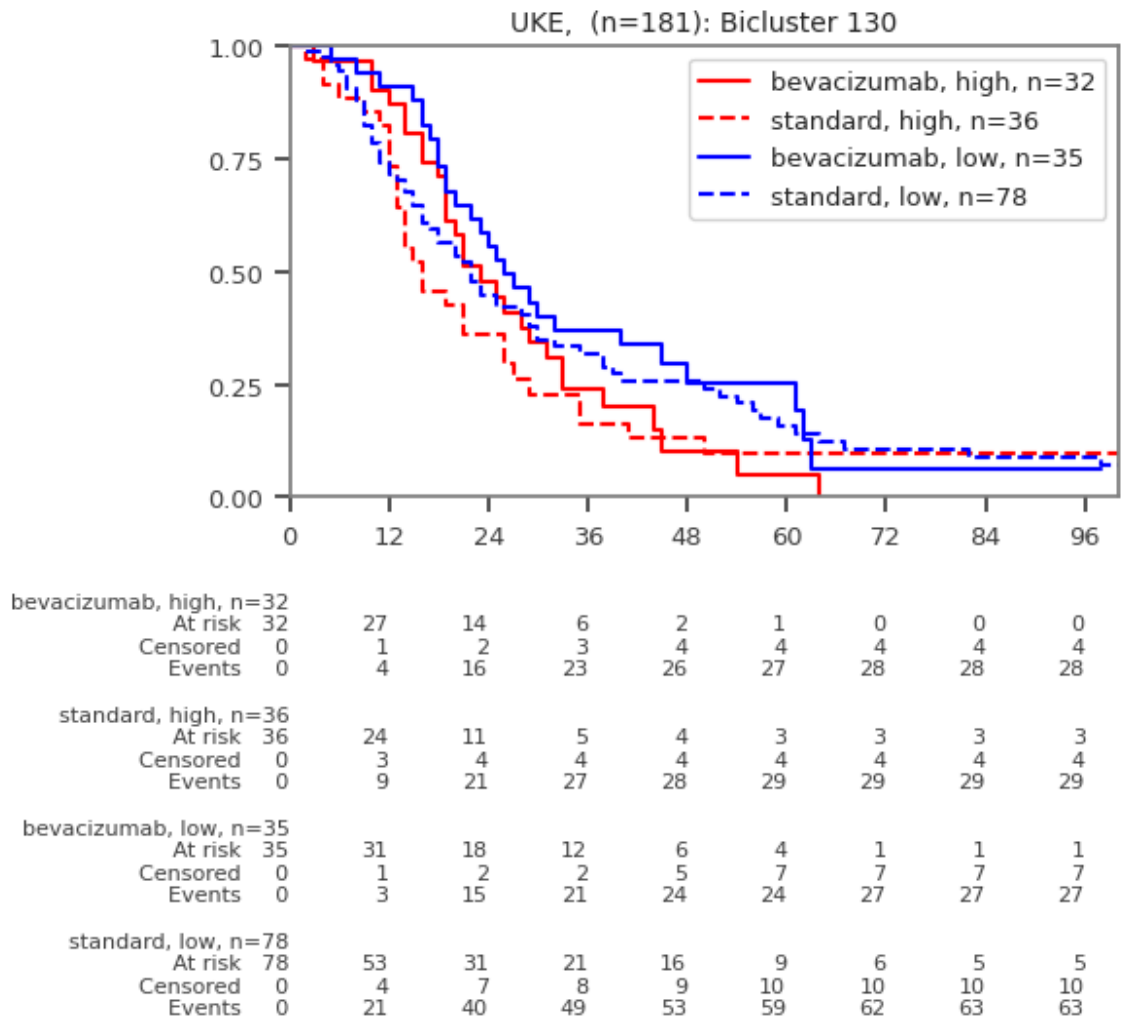

K

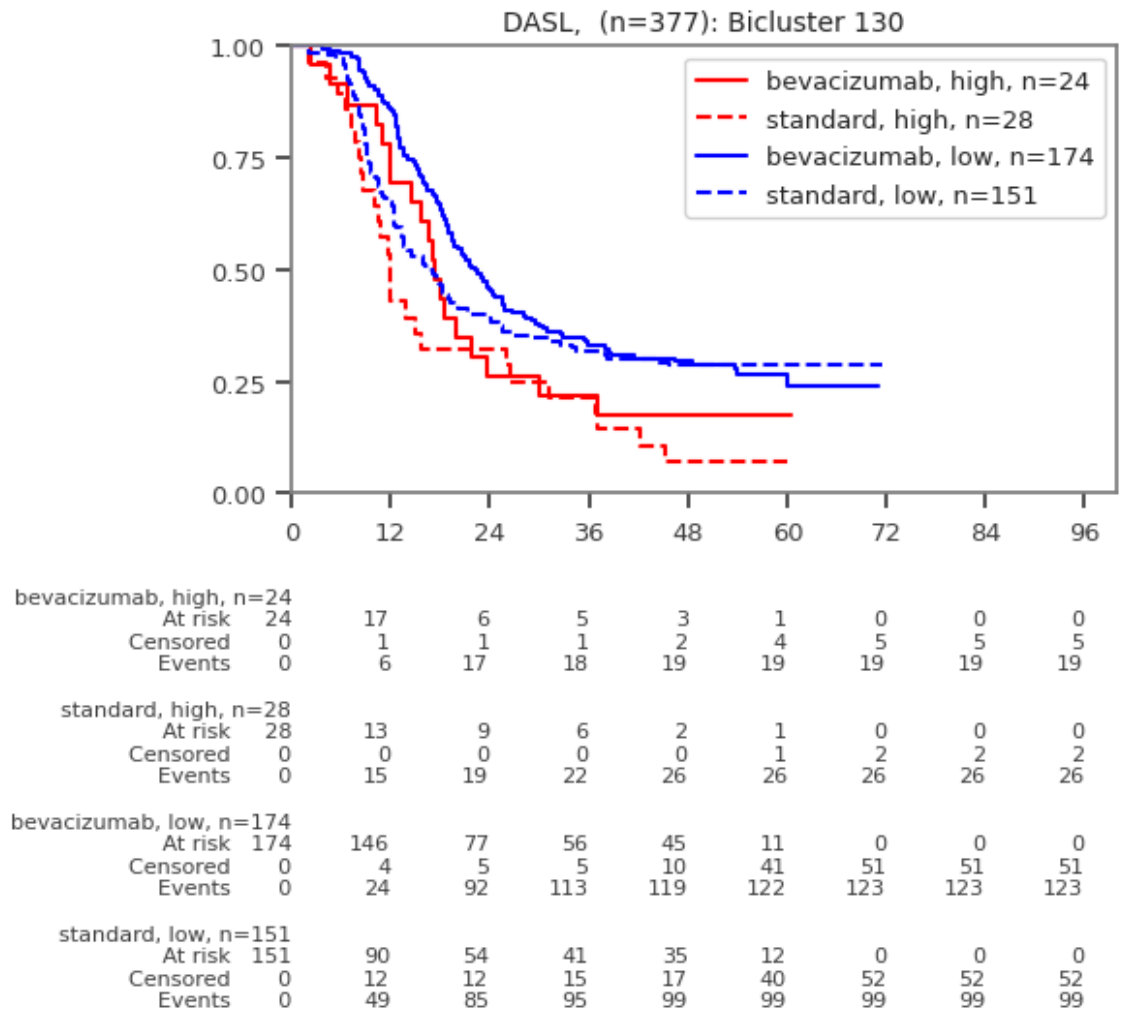

L

Fig. S7. Kaplan-Meier plots for **PFS** of patient subgroups of the UKE and DASL cohorts stratified by treatment (solid line - bevacizumab; dashed line - standard chemotherapy) and expression of genes comprising biclusters 70 (**A,B**),41 (**C,D**), 109 (**E,F**), 95 (**G,H**), and 130 (**K,L**) prioritized by random survival forests as the best predictors of **PFS**.

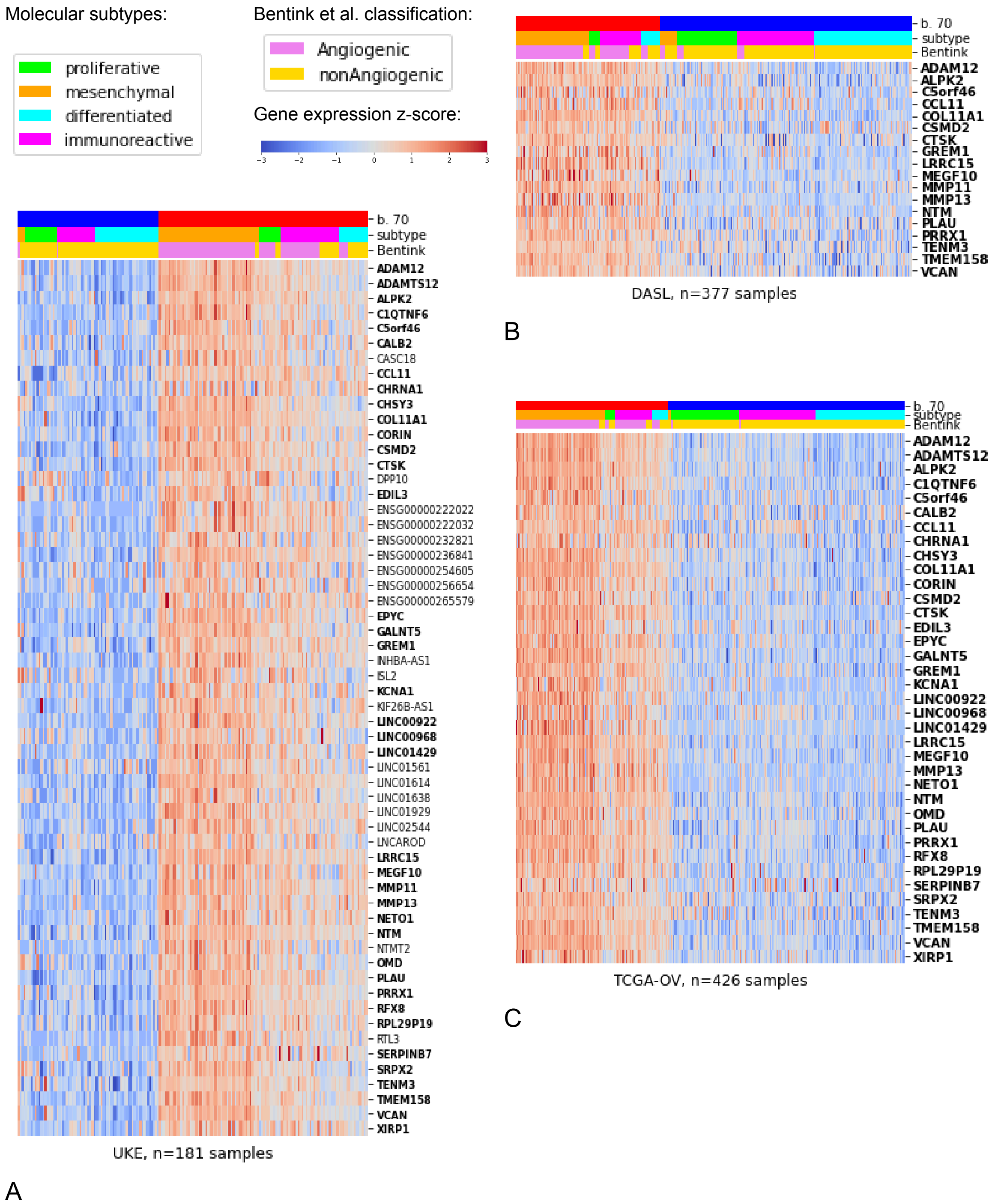

Fig. S8. Tumor stratification by Bicluster 70 is highly correlated with pro-/anti-angiogenic classification defined by Bentink et al. (pro-angiogenic - pink, anti-angiogenic-yellow). **A**. UKE. **B**. DASL **C**. TCGA-OV.

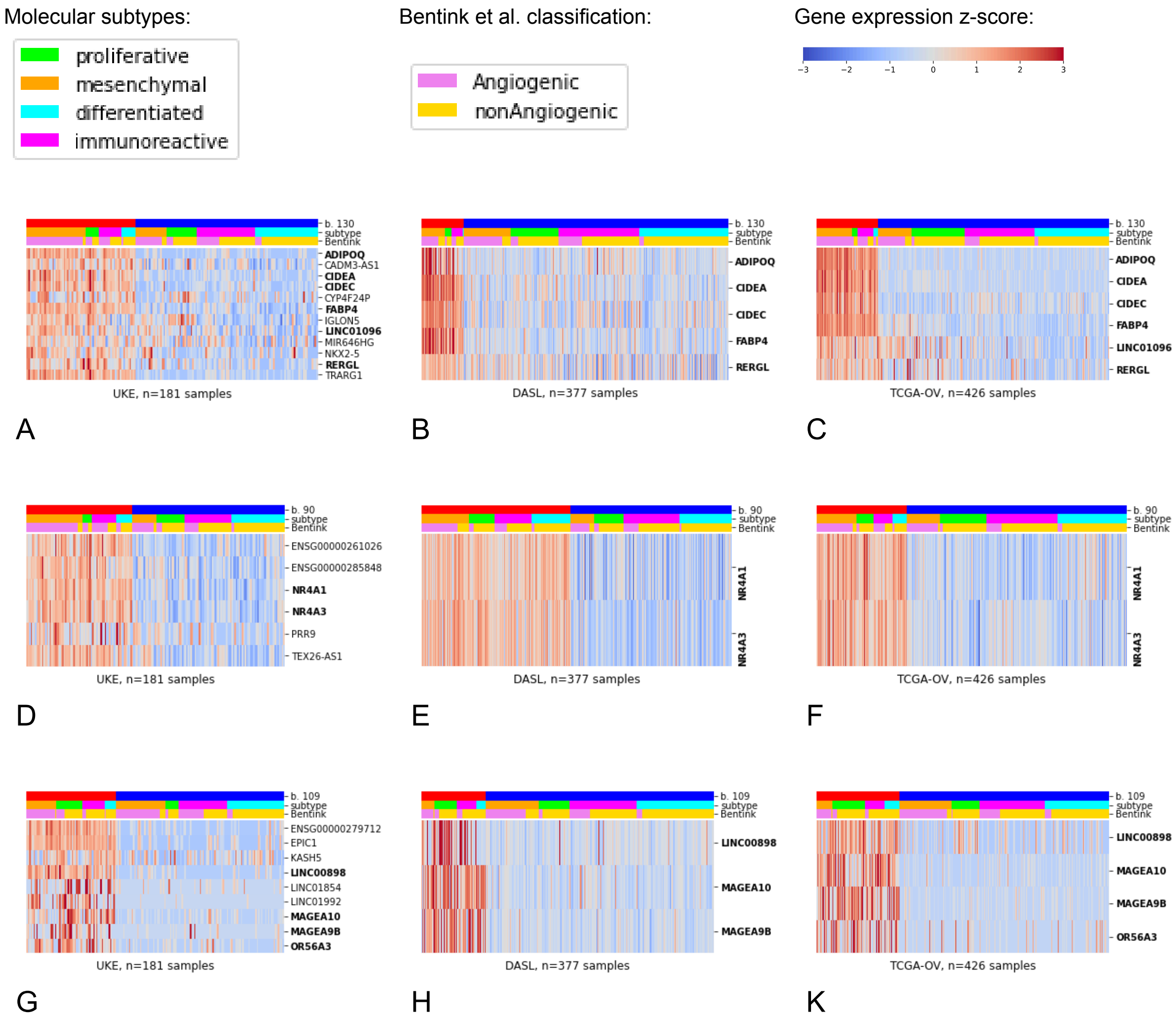

Figure S9. Replication of biclusters in the UKE (**A,D,G**), DASL (**B,E,H**) and TCGA-OV (**C,F,K**) datasets. **A-C**.Bicluster 130. **D-F**. Bicluster 90. **G-K**. Bicluster 109.

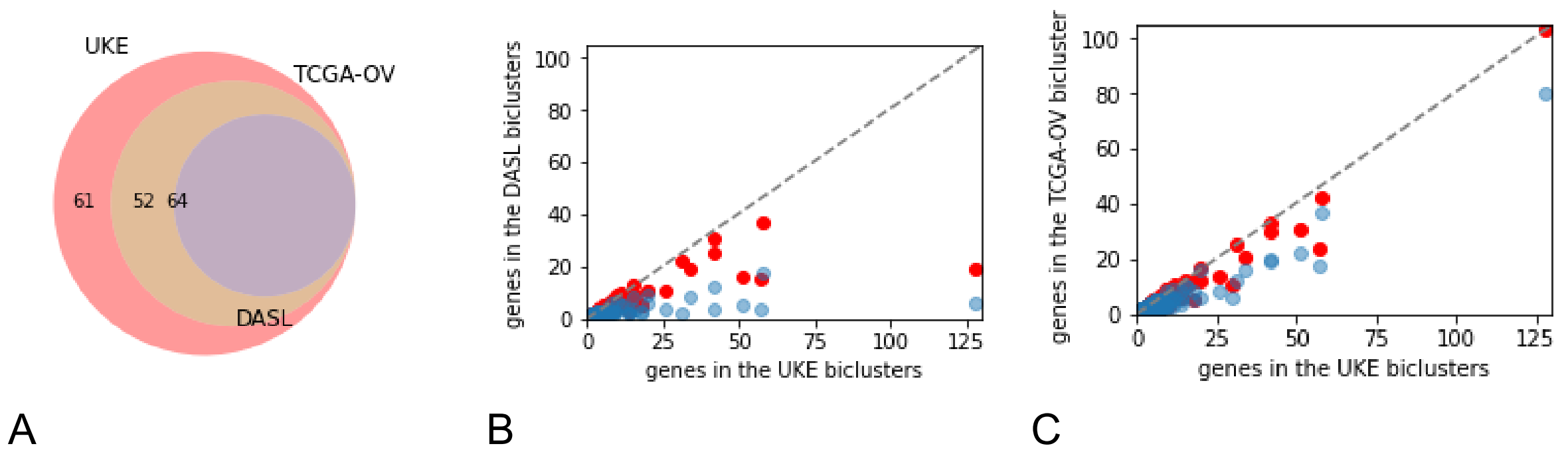

Fig. S10. Replication of biclusters identified in the UKE cohort in the TCGA-OV and in the DASL datasets. **A.** The number of biclusters identified in the UKE cohort (red; n=177 biclusters) and replicated in the TCGA-OV (brown; n=116 biclusters) and in the DASL (violet; n=64 biclusters). For each bicluster discovered in the UKE, in the validation cohort (TCGA-OV (**B**) or DASL (**C**)) were split by 2-means into two clusters based on the expression of bicluster genes. Genes with SNR<0.5 were removed and only biclusters consisting of at least two genes were kept. The number of UKE bicluster genes (x-axis) presented in the replication dataset is shown in red, and the number of genes retained in the bicluster is shown in blue.

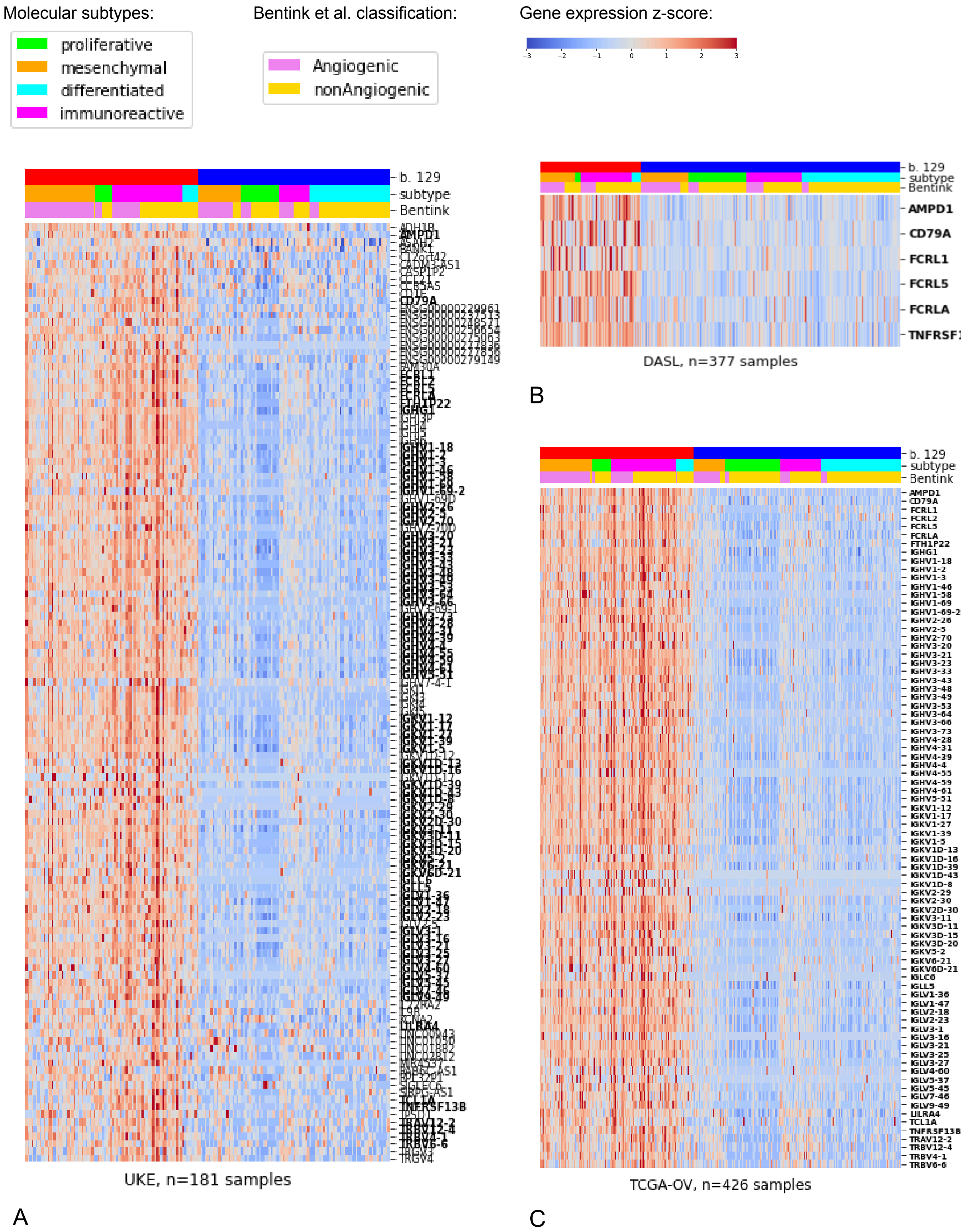

Fig. S11. Bicluster 129 in the UKE (**A**), DASL (**B**) and TCGA-OV (**C**) data.

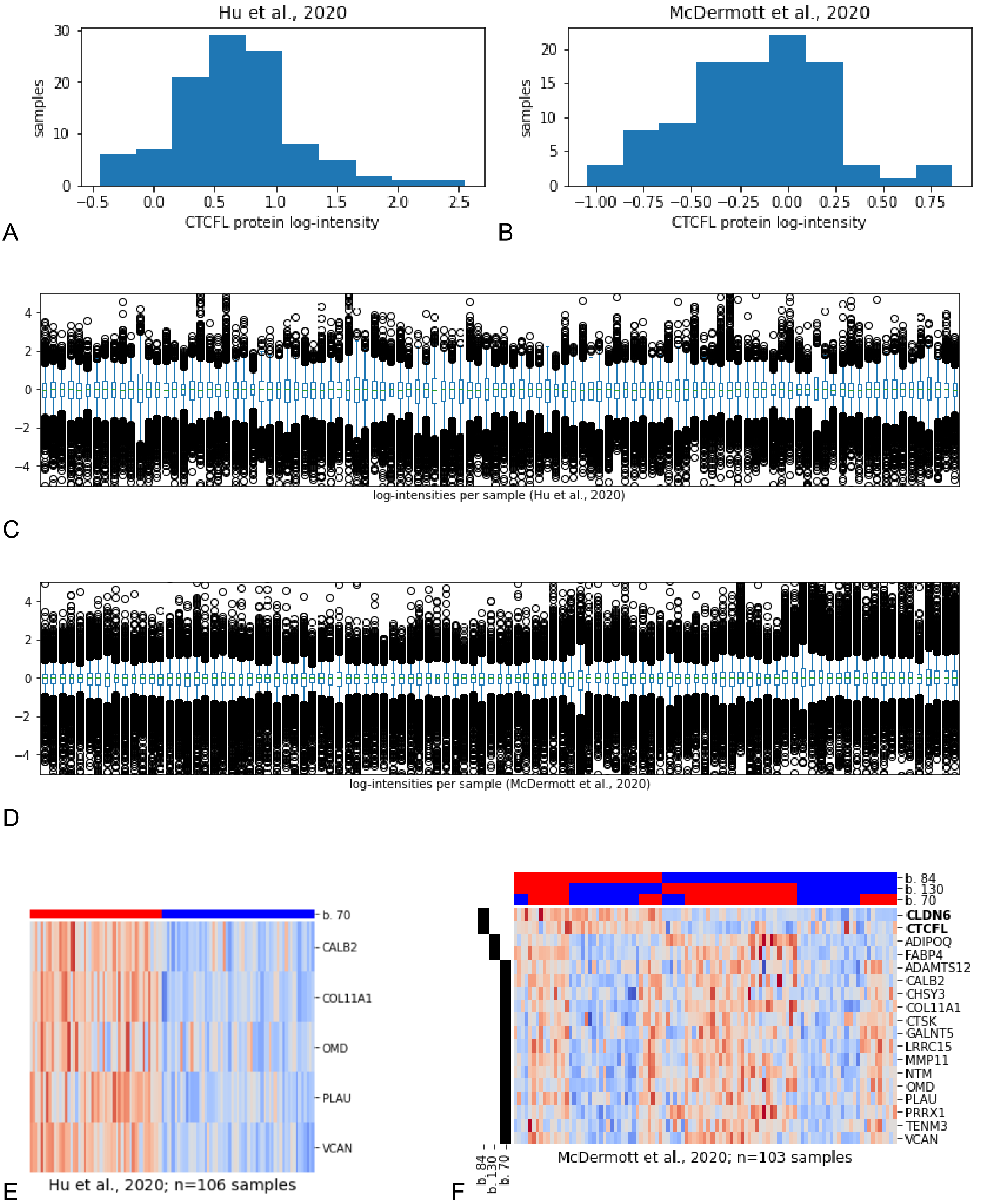

Fig. S12. A-B. The distribution of CTCFL protein expression in two MS-based proteomic datasets from Hu et al. (**A**) and McDermott et al. (**B**). Normalized and log-transformed protein intensities were obtained from supplementary materials. Missing values were replaced with minimal values in the dataset. When multiple measurements were provided for the same protein ids, protein expressions were summarized. **C-D.** Distributions of protein expressions in samples in Hu et al. (**C**) and McDermott et al.(**D**) data**. E-F.** Replication of the selected expression signatures in Hu et al. (**E**) and McDermott et al. (**F**) proteomic datasets.
